# Multivariate canonical correlation analysis identifies additional genetic variants for chronic kidney disease

**DOI:** 10.1038/s41540-024-00350-8

**Published:** 2024-03-09

**Authors:** Amy J. Osborne, Agnieszka Bierzynska, Elizabeth Colby, Uwe Andag, Philip A. Kalra, Olivier Radresa, Philipp Skroblin, Maarten W. Taal, Gavin I. Welsh, Moin A. Saleem, Colin Campbell

**Affiliations:** 1 Intelligent Systems Laboratory, University of Bristol, Bristol, BS8 1TW UK; 2 Bristol Renal, University of Bristol and Bristol Royal Hospital for Children, Bristol, BS1 3NY UK; 3 Department of Metabolic and Renal Diseases, Evotec International GmbH, Marie-Curie-Strasse 7, 37079 Göttingen, Germany; 4 Department of Renal Medicine, Salford Royal Hospital, Northern Care Alliance NHS Foundation Trust, Stott Lane, Salford M6 8HD UK; 5 Centre for Kidney Research and Innovation, University of Nottingham, Derby, UK

**Keywords:** Computational biology and bioinformatics, Genetics, Systems biology, Nephrology, Molecular medicine

## Abstract

Chronic kidney diseases (CKD) have genetic associations with kidney function. Univariate genome-wide association studies (GWAS) have identified single nucleotide polymorphisms (SNPs) associated with estimated glomerular filtration rate (eGFR) and blood urea nitrogen (BUN), two complementary kidney function markers. However, it is unknown whether additional SNPs for kidney function can be identified by multivariate statistical analysis. To address this, we applied canonical correlation analysis (CCA), a multivariate method, to two individual-level CKD genotype datasets, and *metaCCA* to two published GWAS summary statistics datasets. We identified SNPs previously associated with kidney function by published univariate GWASs with high replication rates, validating the *metaCCA* method. We then extended discovery and identified previously unreported lead SNPs for both kidney function markers, jointly. These showed expression quantitative trait loci (eQTL) colocalisation with genes having significant differential expression between CKD and healthy individuals. Several of these identified lead missense SNPs were predicted to have a functional impact, including in *SLC14A2*. We also identified previously unreported lead SNPs that showed significant correlation with both kidney function markers, jointly, in the European ancestry CKDGen, National Unified Renal Translational Research Enterprise (NURTuRE)-CKD and Salford Kidney Study (SKS) datasets. Of these, rs3094060 colocalised with *FLOT1* gene expression and was significantly more common in CKD cases in both NURTURE-CKD and SKS, than in the general population. Overall, by using multivariate analysis by CCA, we identified additional SNPs and genes for both kidney function and CKD, that can be prioritised for further CKD analyses.

## Introduction

Chronic kidney disease (CKD), a major public health burden, affects over 697 million people and causes over one million deaths per year^1^. CKD etiology is complex; its occurrence is related to either a primary renal disorder or a complication of a multisystem disorder or comorbidity (secondary CKD)^2,3^. Estimated glomerular filtration rate (eGFR), used to assess CKD stage, and blood urea nitrogen (BUN or serum urea), are complementary kidney function markers. eGFR is estimated from serum creatinine^4^. BUN measures the nitrogen component of serum urea, the primary metabolite derived from dietary protein and tissue protein turnover^4,5^.

Common genetic variants are thought to contribute to CKD risk via complex genetic architecture^6^. Genome-wide association studies (GWAS) have identified several common SNPs and loci associated with CKD or kidney function^7–19^. In May 2019, a trans-ancestry GWAS meta-analysis of 765,348 CKDGen participants, and replication in 280,722 Million Veteran Program participants, reported 264 eGFR-associated loci (256 for European ancestry), of which 147 (134 for European ancestry) were prioritized as likely relevant for kidney function by additional independent association with BUN^17^. In August 2019, a transethnic GWAS for eGFR in 280,722 Million Veteran Program participants, followed by replication in 765,289 CKDGen participants, confirmed 54 loci and identified 82 previously unreported variants^18^. In 2021, by integrating CKDGen and UK BioBank data (predominantly European ancestry), Stanzick et al identified 424 loci associated with eGFR, of which 348 were classified as likely relevant for kidney function based on additional independent association with either BUN or eGFRcys^19^. Several loci associated with each of eGFR and BUN were identified by the BioBank Japan GWAS (162,255 Japanese individuals)^16^. BUN is affected by other aspects of renal disease, rather than simply filtration rate, but one of its main advantages here is that it is complementary to eGFR^20^. Therefore it has value in validation of SNPs found to be associated with eGFR. A 2021 study integrating GWAS summary statistics with expression quantitative trait loci (eQTL) data, which links SNPs with gene expression in specific cell types, identified over 182 likely causal kidney function genes^21^.

In a typical univariate GWAS, millions of associations between individual genetic variants and a phenotype of interest are tested using regression models^22^. Variants that show a statistically significant association with the trait of interest are typically clustered (due to linkage disequilibrium (LD)) in sets of correlated variants^22^. Canonical correlation analysis (CCA) can simultaneously test for multivariate-based correlation (or co-variance) between multiple SNPs and multiple phenotypic variables^23^. CCA was originally introduced in 1926 to find combinations maximally correlated with each other using linear combinations of variables derived from two sets of data objects^24^. CCA for genotype-phenotype analysis was proposed in 2009^25^ and subsequently extended for testing the association of multiple SNPs with phenotypes in unrelated individuals^26^. CCA is symmetric in that the two datasets have equivalent status, whereas multiple multivariate regression, the most similar statistical method to CCA, is asymmetric in that it tests whether each of the responses can be explained by linear combinations of the explanatory variables. CCA therefore allows the identification of multiple SNPs (gene-gene interactions) and pleiotropic mechanisms thought to be the product of complex genetic diseases^27^. We previously applied CCA to a cardiovascular disease genotype dataset and confirmed already established findings with increased power (*P*-value), and found novel pleiotropic genotype-phenotype associations^23^. *MetaCCA*, developed for identifying multivariate relationships from univariate GWAS summary statistics, has shown agreement with CCA results and identified shared SNPs among type 2 diabetes, obesity and coronary artery disease and stroke risk factors, and between CKD and heart disease^28–31^. However, multivariate methods such as CCA and *metaCCA* have not previously been applied to multiple CKD genomic datasets to look for additional SNPs associated with two kidney function markers, jointly.

Here, to identify additional SNPs associated with two kidney function markers by multivariate methods, we applied CCA and *metaCCA* to two types of CKD genotype-phenotype dataset. We applied *metaCCA* to three publicly available GWAS summary statistics datasets with minor subsets of CKD cases (the CKDGen study (European ancestry) and BioBank Japan)^16,17,28^. We applied CCA to two individual-level SNP genotype datasets of mostly CKD patients (NURTuRE-CKD and the Salford Kidney Study).

## Results

### Single nucleotide polymorphisms identified by *metaCCA*

**Fig. 1:**
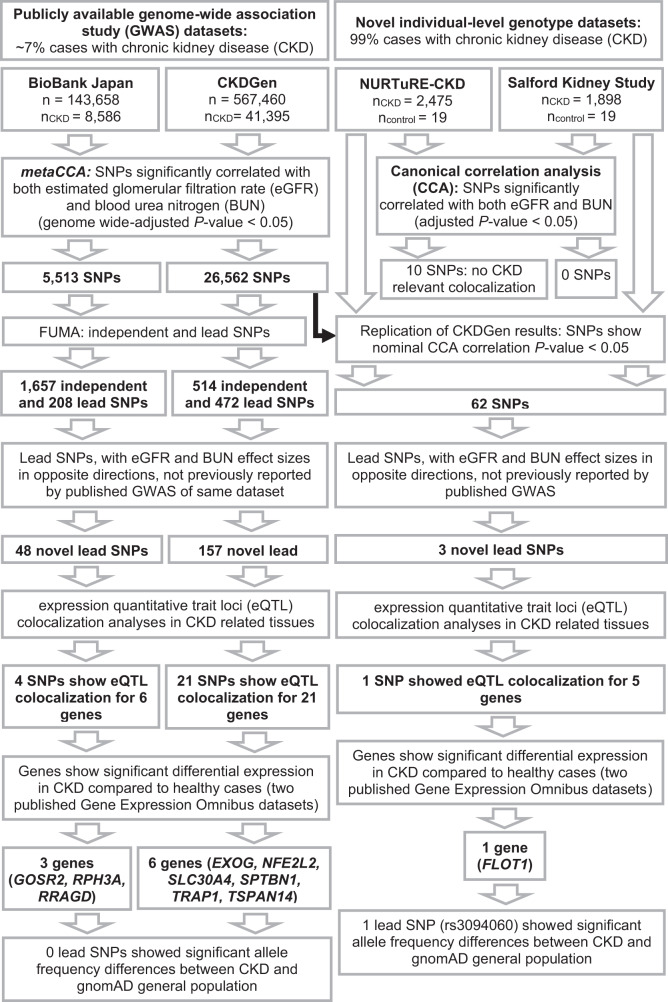
Workflow using *metaCCA* and canonical correlation analysis. Workflow diagram of the analysis method and results obtained for the four datasets.

**Fig. 2:**
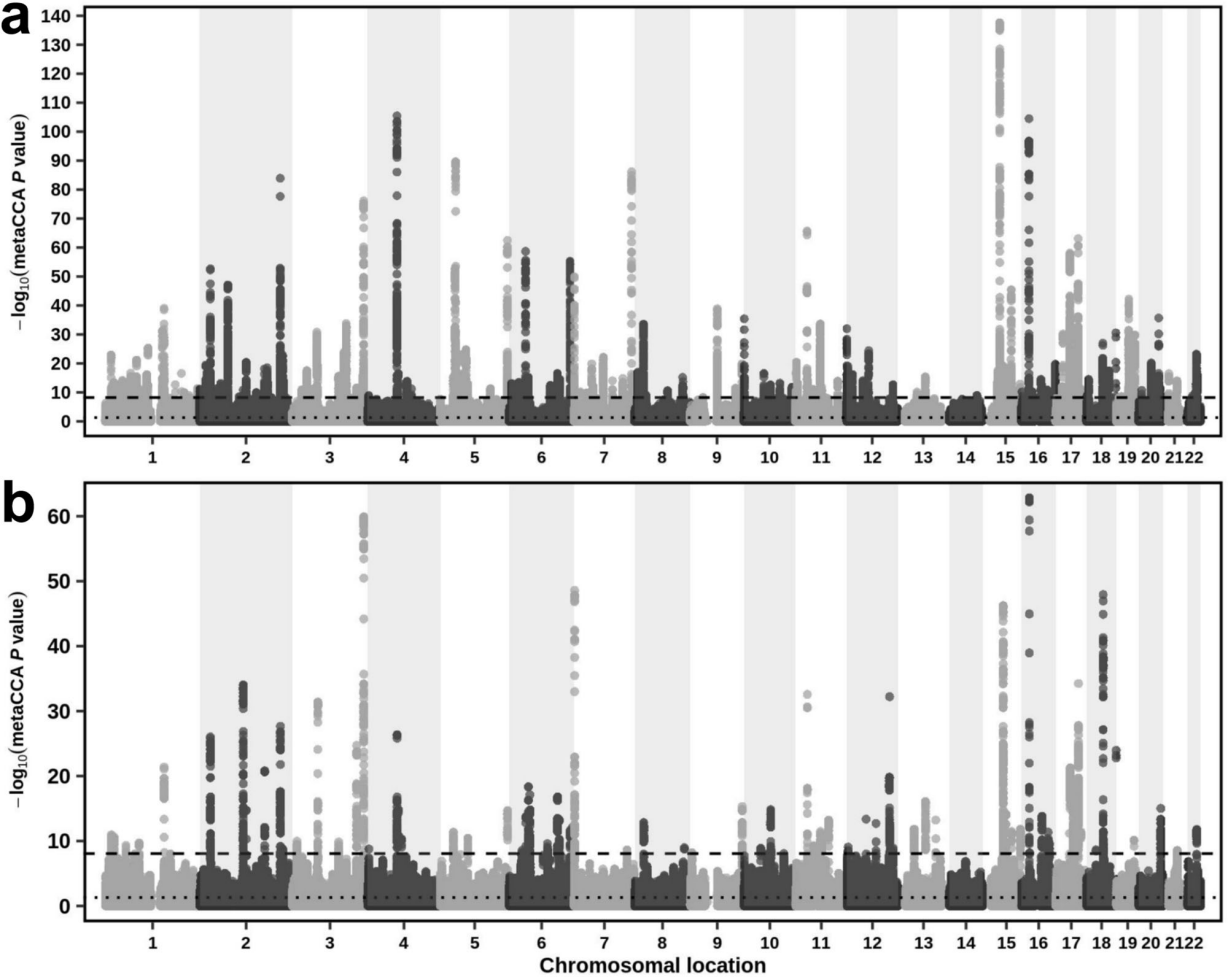
Manhattan plots showing *metaCCA P*-value results for single nucleotide polymorphisms in genome-wide association study datasets. Results for univariate single nucleotide polymorphism (SNP) *metaCCA* applied to (**a**) 8,346,783 CKDGen and (**b**) 5,837,593 BioBank Japan SNPs, to identify correlations with the two phenotypic variables estimated glomerular filtration rate (eGFR) and blood urea nitrate (BUN) considered jointly. Each point represents one SNP with the chromosomal number and co-ordinates on the x-axis and corresponding -log10 *metaCCA P*-value on the y-axis. The dashed line denotes the Bonferroni-corrected *P*-value cut-off of 0.05.

For each of the CKDGen (European ancestry; 567,460 participants) and BioBank Japan (143,658 participants) GWAS summary statistics datasets (for eGFR and BUN), totals of 8,346,783 and 5,837,593 SNPs were analyzed using univariate-SNP *metaCCA* (Table 1, Figs. 1 and 2). Of these, totals of 26,562 (0.3%) and 5513 (0.09%) unique SNPs, respectively, showed a significant correlation with both eGFR and BUN, jointly, using *metaCCA* (Bonferroni-corrected *P*-value < 0.05; Fig. 2). These results reflected their sample sizes (described above) which affected the power to detect SNPs (Table 1). Of these, using the Functional Mapping and Annotation of Genome-Wide Association Studies (FUMA) program, 472 (1.8%) and 208 (3.8%) were lead SNPs (of 514 and 1657 independent SNPs), for totals of 253 and 99 independent genomic loci, respectively (Supplementary Datasets 1 and 2)^32^. Lead SNPs were defined by FUMA as independent SNPs (using LD estimation) with the lowest *P*-value in the genomic region^32^. Of these 472 (1.8%) and 208 (3.8%) identified lead SNPs by *metaCCA* in the CKDGen and BioBank Japan datasets, respectively, totals of 157 (33%) and 48 (23%) lead SNPs were (i) not previously reported as statistically significant for eGFR and BUN by the respective published GWASs^16,17^, and (ii) showed effect sizes in opposite directions (thus compatible for kidney function) (Datasets 1 and 2). These mapped to 117 and 40 independent genomic loci, of which the closest gene annotations for 75 and all 40, had not previously been reported as closest mapped genes for the lead SNPs reported by Wuttke et al.^17^ (CKDGen) and Kanai et al.^16^ (BioBank Japan), respectively (Datasets 1 and 2)^16,17^.

**Table 1:** Datasets analysed in this study

Dataset	Data type	Reference	Number of		
			Participants	Chronic kidney disease cases	Single nucleotide poly-morphisms
NURTuRE-CKD, NURTuRE-controls	Individual-level genotype	NURTuRE Biobank	2519	2500 (99%)	6,419,966
Salford Kidney Study, NURTuRE-controls	Individual-level genotype	Ali et al.^58^	1919	1900 (99%)	6,290,407
CKDGen, European ancestry	Genome-wide association study summary statistics	Wuttke et al.^17^	567,460 (eGFR and BUN)	41,395 (7%)	8,346,783
BioBank Japan	Genome-wide association study summary statistics	Kanai et al.^16^	143,658 (eGFR); 139,818 (BUN)	8586 (5%)	5,961,600

### Overlap with previously reported lead SNPs by published GWAS

**Fig. 3:**
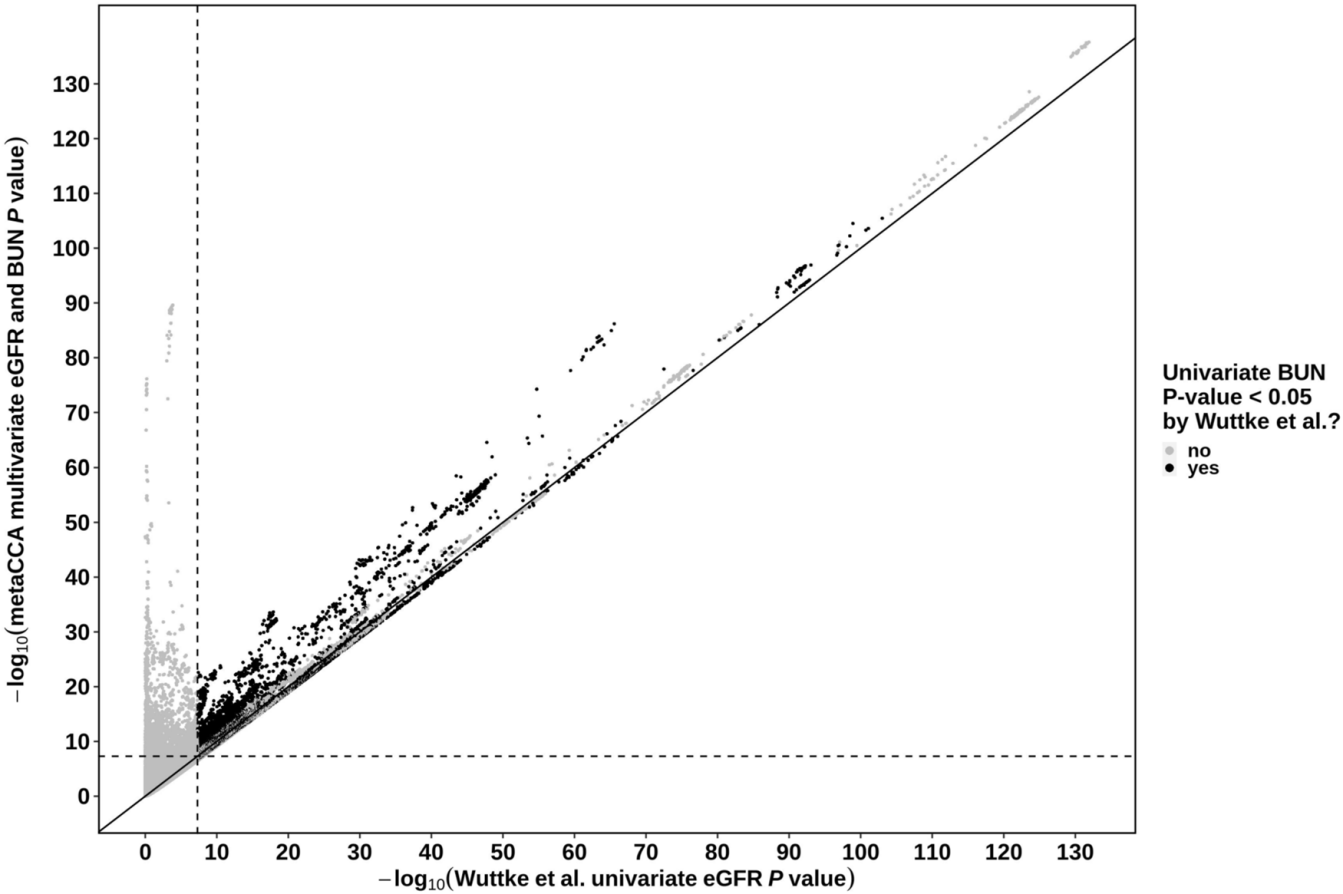
Comparison of CKDGen multivariate *metaCCAP* values with those previously reported by published univariate analysis. Shown are 8,346,783 CKDGen single-nucleotide polymorphisms (SNPs) we analysed using *metaCCA*, which were previously reported to show a significant association with (i) both estimated glomerular filtration rate (eGFR) and blood urea nitrogen (BUN) (black points), or (ii) only eGFR and not BUN (grey points), in the published CKDGen univariate genome-wide association study by Wuttke et al.^17^. The horizontal and vertical dashed lines show the genome-wide statistical significance cut-off equivalent to 0.05 for the *metaCCA* multivariate eGFR and BUN test, and the univariate eGFR test by Wuttke et al.^17^, respectively.

**Fig. 4:**
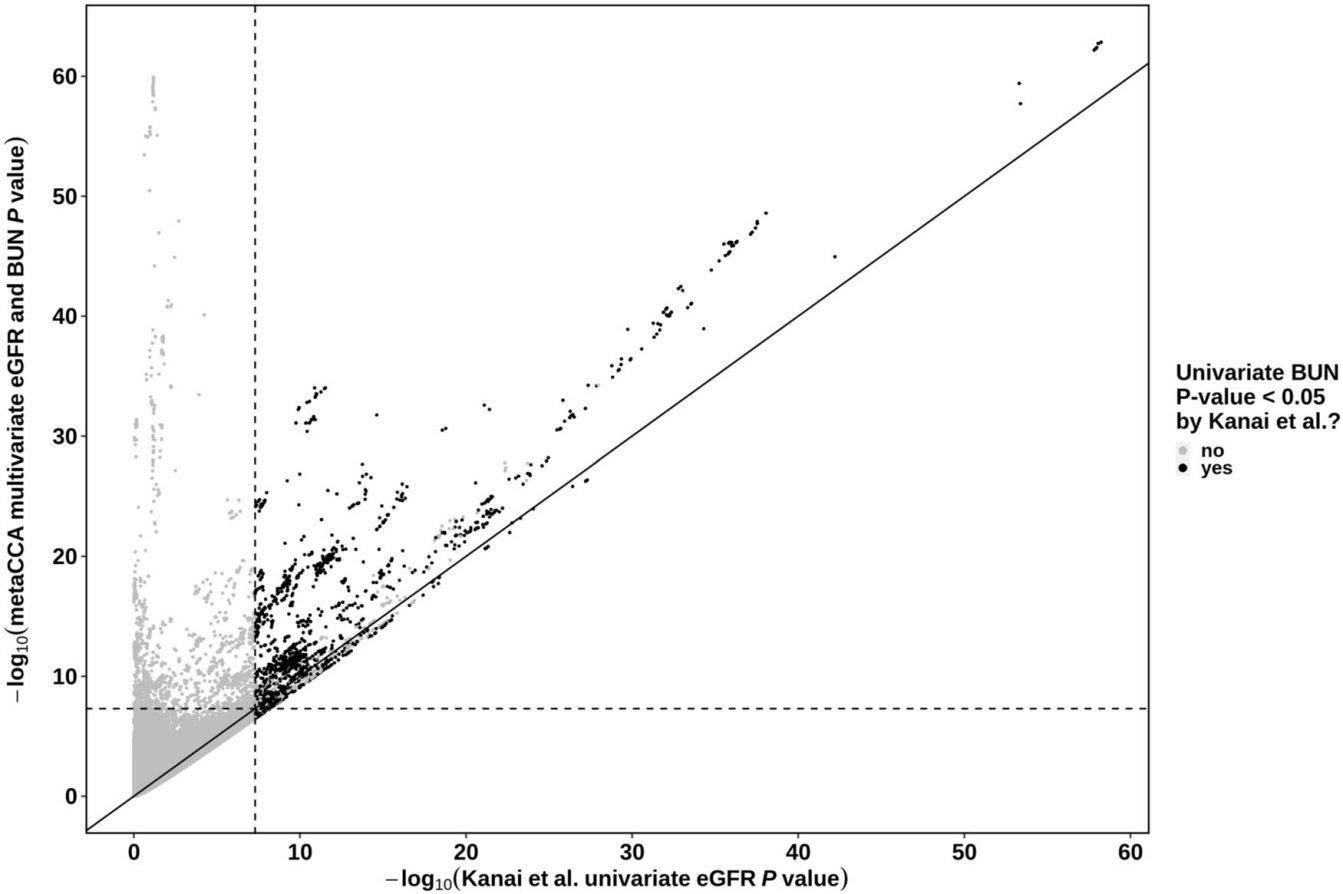
Comparison of BioBank Japan multivariate *metaCCAP* values with those previously reported by published univariate analysis. Shown are 5,837,593 BioBank Japan single-nucleotide polymorphisms (SNPs) we analysed using *metaCCA*, which were reported to show a significant association with (i) both estimated glomerular filtration rate (eGFR) and blood urea nitrogen (BUN) (black points), or ii) only eGFR and not BUN (grey points), in the published Biobank Japan univariate genome-wide association study by Kanai et al.^16^. The horizontal and vertical dashed lines show the genome-wide statistical significance cut-off equivalent to 0.05 for the metaCCA multivariate eGFR and BUN test, and the univariate eGFR test by Kanai et al.^16^, respectively.

For each of the CKDGen and BioBank Japan datasets, we compared *P*-values of *metaCCA*-identified SNPs with those previously reported by the respective published GWAS by using quantile-quantile plots (Figs. 3 and 4). In the published CKDGen GWAS by Wuttke et al., SNPs were defined as significant for kidney function if they showed an association with both eGFR (*P*-value < 5.0 × 10^−8^) and BUN (one-sided *P*-value < 5.0 × 10^−2^), and with eGFR and BUN effect sizes in opposite directions (total of 10,437 SNPs)^17^. For the published BioBank Japan GWAS by Kanai et al.^16^, the eGFR and BUN *P*-value cut-offs were both < 5.0 × 10^−8^ (total of 1241 SNPs with effect size direction filter)^16^. For our *metaCCA* analyses in this paper, we used a standard genome-wide Bonferroni correction (5e−8) to identify significant multivariate *P*-values for both eGFR and BUN (Methods). When compared to the published CKDGen and BioBank Japan univariate GWAS for eGFR and BUN, multivariate eGFR and BUN *metaCCA* identified 9846 (94%), and 1241 (100%) SNPs, respectively, of those previously reported SNPs and identified additional SNPs for kidney function (Figs. 3 and 4). Of the 26,562 (0.3%) CKDGen and 5513 (0.09%) BioBank Japan dataset SNPs that showed a significant correlation with both eGFR and BUN using *metaCCA*, 5840 (22%) and 2471 (45%) SNPs, respectively, had not been previously reported to show a significant association with eGFR and BUN by the published univariate GWASs by Wuttke et al.^17^ and Kanai et al.^16^ (Figs. 3 and 4)^16,17^.

Previously reported lead SNPs that were described as likely relevant for kidney function were listed in Wuttke et al.^17^ for the CKDGen GWAS^17^ and Kanai et al.^16^ for the BioBank Japan GWAS^16^. Of the 122 previously reported lead SNPs in the CKDGen (European ancestry) dataset by Wuttke et al., we found that 113 SNPs (93%) showed a significant correlation with both eGFR and BUN by *metaCCA*, of which 78 SNPs (64%) were defined as lead SNPs using FUMA (Supplementary Dataset 3)^17^. The nine (7%) SNPs missed by *metaCCA* appeared to be due to our use of a Bonferroni correction for the joint eGFR and BUN analyses which was more stringent compared to the GWAS *P*-value cut-off of 0.05 (one-sided) used for the univariate BUN analyses by Wuttke et al.^17^ (Supplementary Dataset 3). We found an overlap of 8/8 (100%) *metaCCA*-identified SNPs with previously reported lead SNPs by the published BioBank Japan GWAS (Supplementary Dataset 4)^16^.

### Overlap of additional *metaCCA*-identified SNPs between CKDGen and BioBank Japan

**Fig. 5:**
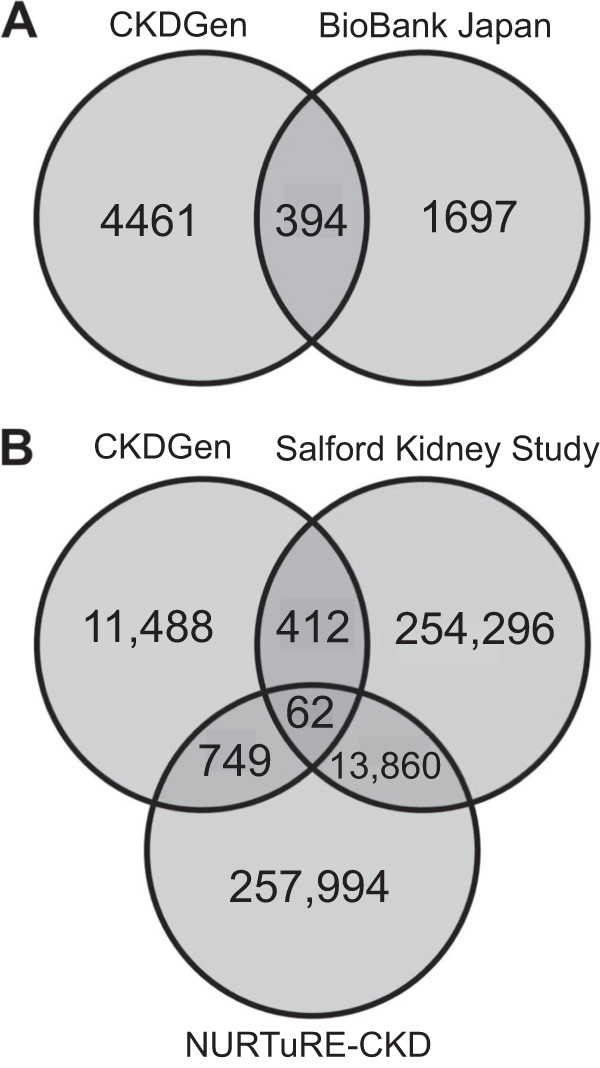
Venn diagrams showing *metaCCA* and canonical correlation analysis-identified single nucleotide polymorphism dataset overlaps. The overlaps in single nucleotide polymorphisms (SNPs) identified for both kidney function variables by (**A**) *metaCCA* for the CKDGen and BioBank Japan datasets, for SNPs not previously reported by the published respective dataset genome-wide association studies (Wuttke et al.^17^ for CKDGen and Kanai et al.^16^ for BioBank Japan), and (**B**) *metaCCA* for the CKDGen dataset and canonical correlation analysis for the NURTuRE-chronic kidney disease and Salford Kidney Study datasets.

Of the previously unreported 5840 CKDGen and 2471 BioBank Japan SNPs for kidney function identified here by *metaCCA*, 4855 and 2091 SNPs, respectively, were available in both datasets, and of these, there was an overlap of 394 (8% and 19%, respectively) SNPs (Fig. 5A, Supplementary Dataset 5). This overlap of 394 SNPs was significant compared to that expected for the number of SNPs analysed using the hypergeometric distribution (*P*-value < 0.05; Table 2). Of these 394 SNPs, using FUMA, 13 (3%) were defined as lead SNPs for 13 independent genomic loci (Supplementary Dataset 5). The canonical correlation coefficients (*r* values) for these SNPs were relatively small, as expected, since multiple small effect common genetic variants in aggregate are thought to be required to show a large enough effect on kidney function and/or CKD risk (Supplementary Fig. 1)^6^.

**Table 2:** Dataset overlap statistics for single nucleotide polymorphisms identified for multivariate kidney function

Intersections between *metaCCA* or canonical correlation analysis identified single nucleotide polymorphisms	Total number of single nucleotide polymorphisms	Degree	Observed overlap	Expected overlap	Fold enrichment	*P-*value
CKDGen (4855) & BioBank Japan (2,091)	5,053,995	2	394	2.897	136.01	2.23E−308
CKDGen (12,718) & NURTuRE-CKD (275,787)	5,410,817	2	812	647.917	1.26	9.54E−11
CKDGen (12,955) & Salford Kidney Study (294,406)	5,845,881	2	487	652.430	0.75	1
CKDGen (12,711) & NURTuRE-CKD (272,655) & Salford Kidney Study (268,630)	5,355,804	3	62	32.457	1.91	2.50E−6

### Additional single nucleotide polymorphisms identified by *metaCCA* and CCA

Next, we investigated whether any of the CKDGen *metaCCA*-identified SNPs that showed eGFR and BUN effect sizes in opposite directions (14,045 of 26,562 SNPs) showed replicated kidney function associations in each of the NURTuRE-CKD (*n* = 2494 including 19 healthy participants) and SKS (*n* = 1917 including a different set of 19 healthy participants) individual-level SNP genotype datasets by using CCA (Fig. 1). Of these 14,045 CKDGen *metaCCA*-identified SNPs, 12,711 SNPs were available for analysis in both the NURTURE-CKD and SKS datasets (Table 2). Of these 12,711 SNPs, 62 (0.5%) SNPs showed nominally significant CCA correlation with both eGFR and BUN in both the NURTuRE-CKD and SKS datasets (*P*-value < 0.05; Table 2, Fig. 5B). This overlap of 62 *metaCCA* and CCA-identified SNPs between the CKDGen (12,711 SNPs), NURTuRE-CKD (272,655) and SKS (268,630) datasets was statistically significant compared to that expected by chance for the number of SNPs analysed by using the hypergeometric distribution (*P*-value < 0.05; Table 2). Of these 62 SNPs (of which rs1398018 and rs9992101 were non-imputed), FUMA analyses showed six lead SNPs which were all imputed SNPs (Supplementary Dataset 6). Of these, three were previously unreported SNP associations for kidney function based on a lack of any published associations for kidney function in the GWAS Catalog or by Wuttke et al.^17^ (Supplementary Dataset 6).

### Colocalisation of *metaCCA*, CCA and eQTL signals

For the four sets of previously unreported lead SNPs identified for kidney function by *metaCCA* and CCA (in CKDGen, in BioBank Japan, in both CKDGen and BioBank Japan, and in CKDGen, NURTuRE-CKD and SKS), to determine whether the genomic signals were colocalised with the primary eQTL signals for closest mapped genes, we assessed colocalisation using the Bayesian test “coloc” (Methods)^33,34^. Colocalisation was defined as a high posterior probability that a single shared variant is responsible for both signals with posterior probability of colocalisation (PP4) ≥ 0.8^33,34^. For eQTL data, we analysed all available datasets in the European Bioinformatics Institute (EBI) eQTL Catalogue and two NephQTL datasets for glomerular and tubular cells^35,36^.

**Fig. 6:**
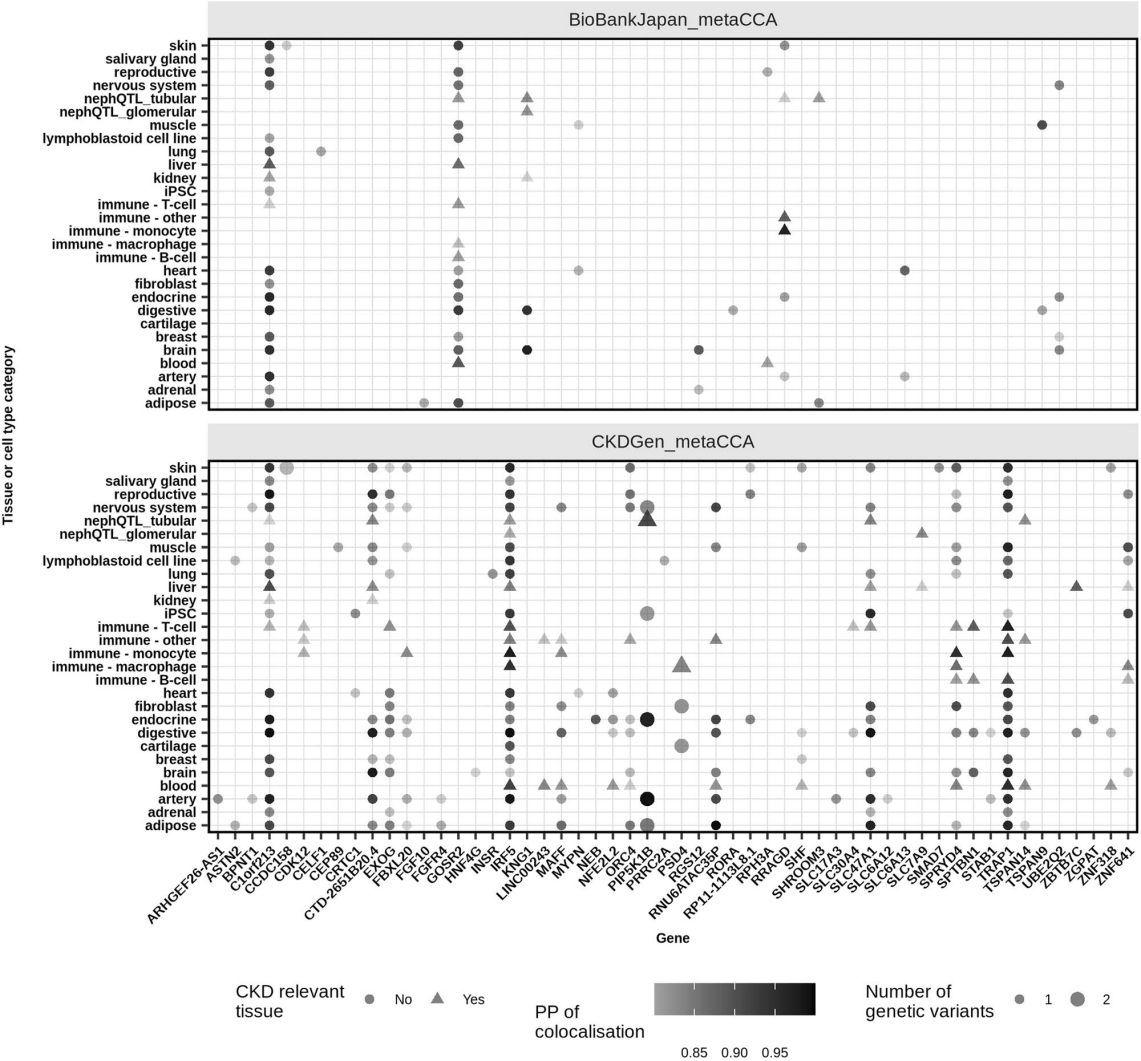
Single nucleotide polymorphism expression quantitative trait loci colocalisation analyses for CKDGen and BioBank Japan *metaCCA* results. For the previously unreported lead single nucleotide polymorphisms identified using *metaCCA* in each of the CKDGen and BioBank Japan datasets, statistically significant colocalisation results using published expression quantitative trait loci datasets, from the European Bioinformatics Institute and NephQTL, are shown by tissue type and gene.

For the 157 previously unreported lead SNPs we identified using *metaCCA* in the CKDGen dataset, a total of 21 (13%) SNPs showed colocalisation for 21 genes in CKD relevant tissues and cell-types (kidney cortex, glomerular or tubular cells, immune cells, liver or blood) and other tissues (Fig. 6, Supplementary Dataset 7). Of these 21 genes, only *CDK12*, *LINC00243* and *SLC7A9* showed colocalisation in only CKD-related tissues or cell-types (Fig. 6).

For the 28 previously unreported lead SNPs we identified using *metaCCA* in the BioBank Japan dataset, a total of four (14%) SNPs showed colocalisation for six genes in CKD relevant tissues or cell-types (Fig. 6, Supplementary Dataset 8).

**Fig. 7:**
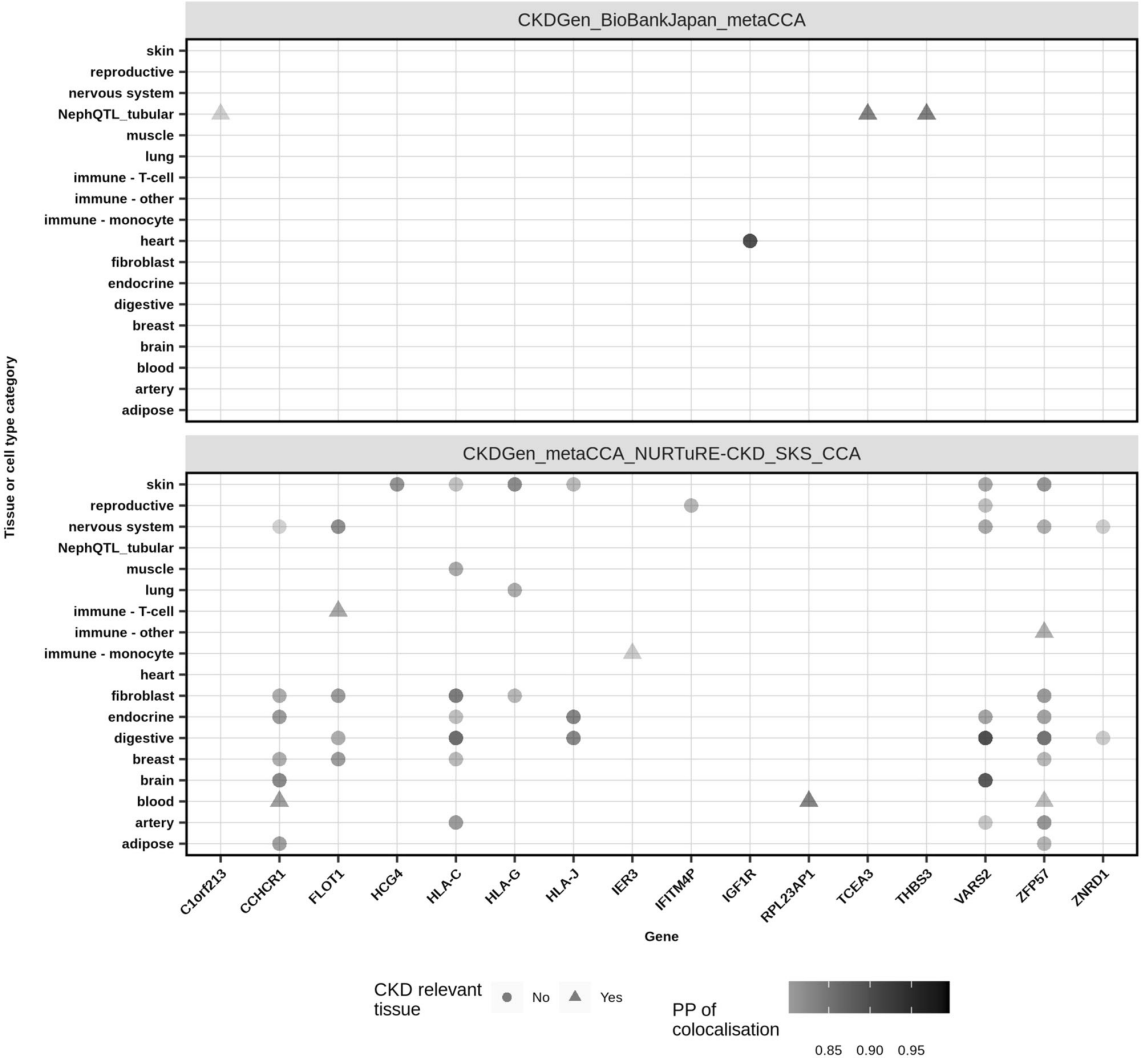
Single nucleotide polymorphism expression quantitative trait loci colocalisation analyses for multiple dataset intersections. For the previously unreported lead single nucleotide polymorphisms (SNPs) identified using *metaCCA* in both the CKDGen and BioBank Japan datasets (13 SNPs), and in the CKDGen, NURTuRE-CKD and SKS datasets (three SNPs), significant colocalisation results using published expression quantitative trait loci datasets, from the European Bioinformatics Institute and NephQTL, are shown by tissue type and gene.

Of the 62 SNPs we identified using both *metaCCA* in the CKDGen dataset and CCA in the NURTuRE-CKD and SKS datasets, one (33%) of the three previously unreported lead SNPs showed colocalisation signals for five genes in CKD relevant tissues and cell-types (Fig. 7, Supplementary Dataset 9). Of these five genes, only two (*IER3* and *RPL23AP1*) showed colocalisation in only CKD-related tissues or cell-types (Fig. 7).

For the 13 previously unreported lead (of 394) SNPs we identified using *metaCCA* in both the CKDGen and BioBank Japan datasets, two (15%) SNPs showed colocalisation signals for three genes in CKD relevant tissues and cell-types (Supplementary Dataset 10). Of these, three genes (*C1orf213, TCEA3, THBS3*) showed colocalisation in only CKD-related tissues or cell-types (Fig. 7).

Overall, for the *metaCCA* or CCA-identifiedpreviously unreported lead SNPs, the fraction of these SNPs that showed colocalisation with their closest mapped genes (loci), thus pointing to a shared underlying SNP associated with both kidney function and gene expression, ranged from 13–33%, as described above. This was greater than the fraction of 16 out of 228 (7%) replicated loci that showed colocalisation reported by Wuttke et al.^17^ for the CKDGen dataset^17^.

### Differential gene expression from published chronic kidney disease datasets

Using the Gene Expression Omnibus to R (GEO2R) web-application, CKD case-control differential gene expression profiles were analyzed for two available published datasets (“expression data from uremic patients and 20 healthy controls”, and “development of gene expression profiles in human chronic kidney disease”)^37–39^. The 21, six, five and three genes that showed colocalisation with *metaCCA*-identified previously unreported lead SNPs in the CKDGen, the BioBank Japan, the CKDGen, NURTuRE-CKD and SKS, and both the CKDGen and BioBank Japan datasets, respectively, were analysed for differential gene expression (Methods). Totals of six (*EXOG*, *NFE2L2, SLC30A4, SPTBN1, TRAP1* and *TSPAN14*), three (*GOSR2, RPH3A, RRAGD*), one (*FLOT1*), and one (*THBS3*) genes, respectively, showed significant log2(fold change) either equal to or greater than 1, or equal to or less than −1, between CKD and healthy controls in these two published gene expression datasets (adjusted *P*-value < 0.05; Supplementary Datasets 11 and 12). This fold change meant that the expression of the gene was increased or decreased in CKD cases relative to healthy cases by a multiplicative factor of at least 2. The *P*-values were adjusted using the default Benjamini and Hochberg false discovery rate method because it provided a good balance between discovery of statistically significant genes and limitation of false positives^39^. In summary, we identified 11 previously unreported lead SNPs for multivariate kidney function that showed eQTL colocalisation with 11 genes which also showed differential expression between CKD and healthy individuals (Table 3). These 11 genes included *TRAP1* which is on the Genomics England congenital anomalies of the kidney and urinary tract gene panel (https://panelapp.genomicsengland.co.uk/panels/234/). These 11 genes showed significant functional enrichment for several processes related to oxidative-stress induced apoptotic signaling pathway and toxin catabolic processes (Benjamini-Hochberg adjusted *P*-value < 0.05, Supplementary Dataset 13).

**Table 3:** Previously unreported lead single nucleotide polymorphisms identified for multivariate kidney function with gene associations

Dataset analysis	Gene symbol	Single nucleotide polymorphism	Gene association method	Published single nucleotide polymorphism univariate GWAS^a^ associations with kidney function (PubMed identifier)
CKDGen	*EXOG*	rs9838792	eQTL^d^ colocalisation	eGFR^e^ (31152163, 33462484)
CKDGen, NURTuRE-CKD^b^ and SKS^c^	*FLOT1*	rs3094060	eQTL colocalisation	none
BioBank Japan	*GOSR2*	rs3851786	eQTL colocalisation	eGFR (35710981), BUN^f^ (31152163, 29403010, 34594039, 36329257)
CKDGen	*NFE2L2*	rs34468415	eQTL colocalisation	eGFR (31152163, 30604766, 31451708, 33462484, 31015462, 35710981, 34272381, 31451708)
BioBank Japan	*RPH3A*	rs11614295	eQTL colocalisation	none
BioBank Japan	*RRAGD*	rs6907843	eQTL colocalisation	eGFR (31152163, 35710981, 34272381), GFR^g^ (29403010)
BioBank Japan	*SLC14A2*	rs1484873, p.Ile132Val	missense variant prediction	eGFR (35710981, 34272381), BUN (31152163, 29403010, 34594039, 36329257, 34272381)
CKDGen	*SLC14A2*	rs41301139, p.Arg896His	missense variant prediction	eGFR (35710981, 34272381), BUN (31152163, 29403010, 34594039, 36329257, 34272381)
CKDGen	*SLC30A4*	rs2453531	eQTL colocalisation	eGFR (33462484)
CKDGen	*SPTBN1*	rs168505	eQTL colocalisation	eGFR (31152163, 30604766, 31152163, 35710981)
CKDGen and BioBank Japan	*THBS3*	rs2974937	eQTL colocalisation	eGFR (33462484, 31451708), BUN (34594039, 31152163)
CKDGen	*TRAP1*	rs1635404	eQTL colocalisation	eGFR (31152163, 31451708, 35710981, 34272381)
CKDGen	*TSPAN14*	rs7087356	eQTL colocalisation	eGFR (31152163, 35710981, 34272381), BUN (34272381)

^a^Genome-wide association study.

^b^Chronic kidney disease.

^c^Salford Kidney Study.

^d^Expression quantitative trait loci.

^e^Estimated glomerular filtration rate.

^f^Blood urea nitrogen.

^g^Glomerular filtration rate.

### Chronic kidney disease allele frequency analyses

For the 11 previously unreported *metaCCA* and/or CCA identified lead SNPs that showed colocalisation with 11 differentially expressed genes in CKD, SNP allele frequencies in CKD cases in each of the NURTuRE-CKD and SKS datasets were computed and compared to gnomAD (general population) allele frequencies (Supplementary Dataset 14)^40^. Of these 11 SNPs, only one SNP (rs3094060), which colocalised with *FLOT1* gene expression, showed a significant difference greater than 15% in allele frequency (AF) between the gnomAD general population (non-Finnish European), and the same type of CKD cases in both the NURTuRE-CKD and SKS datasets (Chi-square test with Yates’ correction *P*-value < 0.05; Supplementary Dataset 14)^40^. This was observed for the membranous nephropathy (MN) CKD cases only (Supplementary Dataset 14).

### Previously unreported lead missense single nucleotide polymorphisms identified using *metaCCA*

Of the 157 and 28 previously unreported lead SNPs identified in each of CKDGen and BioBank Japan, six and five, respectively, encoded missense variants, of which missense variants in *SLC14A2* were identified in both datasets (Supplementary Dataset 15). Of these two missense SNPs, p.Arg896His identified in CKDGen was predicted to be “pathogenic”, “deleterious” and “probably damaging” by several in silico variant prediction tools (also with a high CADD score) which suggested a negative effect on protein structure and function (Supplementary Dataset 15). However, neither of these *SLC14A2* missense variants showed a difference greater than 15% in allele frequency (AF) between the gnomAD general population (non-Finnish European) and the same CKD cases in both the NURTuRE-CKD and SKS datasets (Supplementary Dataset 14). *SLC14A2* was not previously reported for both eGFR and BUN by the published GWASs^16,17^.

## Discussion

GWASs have identified several common SNPs and loci associated with CKD or kidney function biomarkers^7–19^. GWAS methods typically measure the association between each SNP and one phenotype using regression methods^22^. Multiple small effect common genetic variants may contribute to kidney function and/or CKD risk, in a polygenic manner^6^. CCA can identify joint correlations between multiple SNPs and multiple phenotypic variables simultaneously^23^. By this, CCA allows the identification of multiple SNPs (gene-gene interactions) and pleiotropic mechanisms, which are thought to be the product of complex genetic diseases^27^. Earlier studies suggest greater power can be achieved by using CCA or *metaCCA*, leading to novel findings^23,28^. Multivariate methods such as CCA and *metaCCA* have not previously been applied to genomic datasets with either major and minor CKD subsets to look for additional SNPs associated with two kidney function markers, jointly.

In this study, to identify SNPs associated with multivariate kidney function (both eGFR and BUN), we applied CCA to two individual-level CKD genotype datasets (NURTURE-CKD and SKS) and *metaCCA* to two GWAS summary statistics datasets, CKDGen and BioBank Japan. These CKDGen and BioBank Japan datasets each had a minor subset of CKD cases. For all four datasets, baseline eGFR and BUN measurements or GWAS summary statistics were available. We identified several previously unreported replicated SNPs that showed significant correlation with both eGFR and BUN using CCA and *metaCCA*. We compared our findings with published univariate-GWAS SNPs, assessed replication across datasets, eQTL colocalisation, differential gene expression between CKD cases and healthy individuals, allele frequency analyses, and missense variant effect predictions.

For the two GWAS summary statistics datasets, using univariate-SNP *metaCCA*, we identified many SNPs that showed a significant correlation with both eGFR and BUN jointly. Of the 122 previously reported lead SNPs for both eGFR and BUN in the published CKDGen GWAS (European ancestry)^17^, our *metaCCA* results showed a high replication rate of 93%. For the 7% (nine) missed SNPs, this was likely due to the stricter *P*-value threshold we used for *metaCCA*. For the BioBank Japan dataset, we found 100% overlap between our *metaCCA*-identified SNPs with the eight previously reported lead SNPs by the published BioBank Japan univariate kidney function GWAS^16^. Overall, this showed that *metaCCA* had successfully identified previously reported SNPs associated with kidney function in the same datasets.

In the eQTL colocalisation analyses, in addition to kidney cell-types, the immune system was also seen as a CKD relevant cell type to include because some subsets of lymphocytes produce cytokines that can induce or reduce renal inflammation^41^. Furthermore, several human leukocyte antigens encoded at the major histocompatibility complex are associated with increased or decreased risk of renal failure^42^, and low levels of some T-cell subsets in peripheral blood were associated with renal outcome in CKD^43^. We also included the liver as CKD-relevant because some genetic diseases are associated with both kidney and liver disease, e.g. hepatorenal fibrocystic disease^44^.

Across all four *metaCCA*/CCA analyses (the CKDGen, the BioBank Japan, both CKDGen and BioBank Japan, and the CKDGen, NURTuRE-CKD and SKS analyses), we identified a total of 11 genes (*EXOG, FLOT1, GOSR2, NFE2L2, RPH3A, RRAGD, SLC30A4, SPTBN1, THBS3, TRAP1, TSPAN14*) that colocalised with the identified 11 previously unreported lead SNPs for multivariate kidney function, in CKD-relevant tissues, and also showed differential expression between CKD and healthy individuals in two published GEO datasets. These 11 genes showed significant functional enrichment for the negative regulation of oxidative stress-induced intrinsic apoptotic signaling pathway. Inflammatory cytokines associated with oxidative stress promote the damage of renal tissues by inducing apoptosis, necrosis, and fibrosis and may play an important role in the pathogenesis and progression of CKD^45,46^. These 11 genes included *TRAP1* (tumor necrosis factor receptor-associated protein 1) which is on the Genomics England congenital anomalies of the kidney and urinary tract gene panel (https://panelapp.genomicsengland.co.uk/panels/234/).

Replicated in both the CKDGen and BioBank Japan datasets, we identified a total of 394 previously unreported SNPs for both eGFR and BUN (multivariate kidney function) by using *metaCCA*. Using the hypergeometric statistical test, this SNP overlap between the European and Japanese population datasets was significantly greater than that expected by chance for the numbers of SNPs analysed. Of these 394 SNPs, 13 were lead SNPs for 13 independent genomic loci, of which two SNPs showed colocalisation signals for three genes, in CKD relevant tissues and cell-types only. Of these three genes, the *THBS3* gene also showed significant differential gene expression between CKD and healthy controls in published gene expression datasets. This suggested that SNPs affecting *THBS3* expression were associated with both kidney function and CKD. However, we did not find any significant association of the identified *THBS3* lead SNP (rs2974937) with CKD in our two European ancestry CKD cohorts using AF analyses, compared to the gnomAD non-Finnish European ancestry general population. In the GWAS Catalog, *THBS3* had previously been associated with univariate kidney function by other GWAS by other SNPs, but not both eGFR and BUN in the same study^47^. This could be due to the SNP only affecting a subset of CKD patients, for example tubular nephropathies with certain features, that we did not analyse, or that the SNP may only show a significant AF difference compared to the general population when analysed in aggregate with multiple SNPs (polygenic complex genetics). Another reason could be that, although the kidney function associated SNP colocalised with and so appears to affect *THBS3* expression, the *THBS3* expression changes seen in CKD could be an effect of CKD rather than a cause. Mendelian Randomisation studies would be needed to investigate whether *THBS3* expression changes are on the causal pathway between the SNP and CKD and could include testing a broader range of SNPs in/associated with *THBS3*. In addition, further AF analyses in individual-level genotype datasets for Japanese and East Asian populations would be needed.

Observed in the three European ancestry datasets (CKDGen using *metaCCA* with a genome-wide *P*-value cut-off, and NURTURE-CKD and SKS datasets using CCA with a nominal *P*-value cut-off), were 62 SNPs, which showed a significant overlap compared to that expected by chance for the number of SNPs analysed using the hypergeometric distribution. Of these 62 SNPs, FUMA analyses showed six lead SNPs, of which three SNPs were previously unreported kidney function associations. Of these, one SNP showed colocalisation signals for five genes in CKD relevant tissues and cell-types. Of these five eQTL genes, *FLOT1* showed significant differential gene expression between CKD and healthy controls in published gene expression datasets from the Gene Expression Omnibus. Furthermore, the lead SNP (rs3094060) identified for *FLOT1* also showed a significant difference in AF between CKD MN cases and gnomAD, in both NURTURE-CKD and SKS. This suggested that this SNP was associated with both kidney function and MN CKD sub-type by affecting *FLOT1* expression*. FLOT1* is a scaffold protein of lipid rafts and is involved in several biological processes including lipid raft protein‑dependent and clathrin‑independent endocytosis, and may also play a role in immune T-cell activation and cell adhesion^48^. Using microarray gene expression network analyses, *FLOT1* was also identified as a potential target regulated by core transcription factors related to the immunoreaction in nocturnal hemodialysis treatment in end stage renal disease patients^49^. These reported potential immune system involvements of *FLOT1* would align with clinical features of membranous nephropathy which is an auto-immune type of kidney disease. In summary, using CCA and *metaCCA*, with eQTL colocalisation and differential gene analyses, we identified a previously unreported kidney function SNP which colocalised with *FLOT1* expression and was significantly more common in MN CKD patients compared to the general population. Overall, this suggested that this SNP may contribute to MN manifestation by affecting *FLOT1* gene expression, possibly by immune system perturbation. *FLOT1* has not previously been associated with kidney function from GWAS in the GWAS Catalog database thus was considered a previously unreported gene association for kidney function, along with the colocalised rs3094060 lead SNP.

For each of the CKDGen and BioBank Japan datasets, although not replicated SNPs, with the addition of orthogonal datasets including eQTL (in CKD-relevant tissues) and CKD gene expression datasets (from the GEO), we also identified nine previously unreported lead SNPs that showed colocalisation with nine genes. These nine genes (*EXOG, NFE2L2, SLC30A4, SPTBN1, TRAP1* and *TSPAN14* in CKDGen, and *GOSR2, RPH3A*, and *RRAGD* in BioBank Japan) showed significant differential expression in CKD compared to healthy controls. *RPH3A* lacked any kidney function associations in the GWAS Catalog, thus appeared to be a previously unreported SNP finding for kidney function. *RPH3A* expression was enhanced in several human proteinuric diseases, and was also altered in mouse and human proteinuric disease^50^. Furthermore, combined genomic and metabolomic analyses have previously associated increased urinary albumin excretion in the general population with *RPH3A* polymorphism^51,52^. However, none of the lead SNPs identified for these nine genes showed AF differences greater than 15% between CKD cases (in each of the NURTURE-CKD and SKS) and the general population gnomAD dataset. This was despite showing eQTL colocalisation with genes in CKD-relevant tissues which also showed significant differential gene expression between CKD and healthy controls in published gene expression datasets. Possible reasons for these observations are the same as those described above for the *THBS3* lead SNP.

In addition, we identified a total of 11 previously unreported lead missense SNPs, of which missense variants in *SLC14A2* were seen in both the CKDGen and BioBank Japan dataset *metaCCA* analyses. Of these two SLC14A2 variants, one (p.Arg896His) was predicted as “likely pathogenic”, “deleterious” and “probably damaging” by several in silico variant prediction tools (with a high CADD score). This suggested p.Arg896His may affect kidney function in both European populations by altering SLC14A2 protein structure and/or function. *SLC14A2* was not previously reported for both eGFR and BUN by the published univariate kidney function GWASs, but has been associated with eGFR and BUN in other studies in the GWAS Catalog^16,17^. SLC14A2 is a urea transporter which plays an important role in urine concentration; in knockout mice lacking urea transporters, urine volumes were increased^53,54^. Volume overload is a risk factor for mortality in CKD and end stage renal disease patients^55,56^. However, neither of these two identified *SLC14A2* missense SNPs showed AF differences greater than 15% between CKD cases (in each of the NURTURE-CKD and SKS) and the general population gnomAD dataset. Again, possible reasons for this are described above for the *THBS3* lead SNP.

In the NURTURE-CKD dataset, we also identified a further 10 SNPs that showed genome-wide significant CCA correlation with both baseline eGFR and BUN, jointly. However none of these SNPs showed colocalisation with genes in CKD-relevant tissues thus were not investigated further.

In summary, by applying a multivariate statistical approach, CCA, to four independent CKD datasets, we identified both previously reported and unreported SNPs associated with kidney function, beyond those reported by published univariate-trait GWAS methods. We identified a total of 11 previously unreported lead SNPs that showed eQTL colocalisation with 11 genes in CKD relevant tissues, that also showed significant differential expression between CKD and healthy individuals. Of these, two genes (*FLOT1* and *RPH3A*) were previously unreported SNP kidney function gene associations. Furthermore, the SNP colocalised with *FLOT1* gene expression (rs3094060) showed significant association with MN CKD cases in both NURTURE-CKD and SKS using AF analyses. Overall, by using multivariate analysis by CCA, we identified several previously unreported SNPs and genes for both kidney function and CKD, that can be prioritized for further CKD analyses.

There were some limitations to our study. Firstly, it was possible that identified SNPs were merely correlated with the risk-modifying variants, as with other genome-wide tag-SNP array datasets. However, by adding orthogonal information on the SNPs such as published eQTL SNP data, as well as the FUMA SNP independence tool, this provided additional supportive evidence of likely functional SNPs for further investigation. Secondly, since serum urea is affected by dilution/concentration of plasma (volume status), diet on a day-to-day basis and treatment, it is not as reliable an estimator of kidney function as eGFR. However, since it is complementary to eGFR^20^, it has value in validation of kidney function SNPs found to be associated with eGFR. Thirdly, the NURTuRE-CKD and SKS datasets were considerably smaller than the CKDGen and BioBank Japan datasets, thus affecting power to detect, however power analyses showed it was theoretically possible to detect small effect SNPs using CCA. It was not possible to amalgamate the NURTURE-CKD and SKS datasets with the GWAS summary statistics datasets as they were of different data-types (individual level genotype data and GWAS summary statistics data, respectively). Finally, all three GWAS summary statistics datasets may have also contained SNPs with some missing genotype data beyond the GWAS quality control checks reported in the published studies. These factors may have lead to effect size variability between the datasets.

## Methods

### NURTuRE-CKD and Salford Kidney Study participants

The NURTuRE-CKD cohort has linked genotype-phenotype data, and all participants provided written informed consent^57^. The study was approved by the South Central—Berkshire Research Ethics Committee, abides by the principles of the Declaration of Helsinki and is registered at ClinicalTrials.gov (NCT04084145)^57^. The Salford Kidney Study (SKS) dataset, which has been described previously, received ethical approval from the North West Greater Manchester South Research Ethics Committee (REC15/NW/0818) and written informed consent was obtained from all patients^58^. For the genome-wide genotyping, blood samples were collected, stored as whole blood or centrifuged to separate plasma or serum, aliquoted and frozen at −80 °C. Deoxyribonucleic acid was extracted from the frozen whole blood. Of the NURTuRE cohort, 2903 CKD and 99 control participants were genotyped^57^. Of the Salford Kidney Study (SKS) cohort, 2409 participants were genotyped^58^. NURTuRE-CKD and SKS cohort participants had non-dialysis dependent CKD since they were recruited if their eGFR was below 60 ml/min/1.73 m^2^ (all SKS patients) or if urine albumin to creatinine ratio was >30 mg/mmol in those with eGFR above 60 ml/min/1.73 m^2^. End-stage kidney disease and renal replacement therapy were exclusion criteria.

### Single nucleotide polymorphism genotype data processing

For each of the NURTuRE (including 99 controls) and SKS datasets, SNPs were genotyped using the Illumina Global Screening array v2.0 with additional multi-disease content and 2k custom sequences. After excluding duplicated probes using BCFTools version 1.9 (using htslib 1.9), totals of 671,485 and 672,412 variants remained, respectively^59^. The following steps were carried out using Plink version 2^60^. Variants were excluded if the minor allele frequency (MAF) < 0.01 (162,483 and 170,554 variants excluded, respectively), missing SNP genotype call rate ≥1.5% (19,149 and 23,889 SNPs excluded, respectively), Hardy-Weinberg assumptions violated with *P*-value < 0.001% (116 and 71 SNPs excluded, respectively) and if they were located on mitochondrial or sex chromosomes (21,133 and 19,426 SNPs excluded, respectively). From the SKS dataset only, a total of 135 overlapping samples with NURTURE-CKD were excluded. Further samples were excluded if they were known first or second-degree relatives of another participant (13 and 0 excluded), showed gender mismatches (11 and 33 excluded), showed >10% low call rate SNPs (12 and 32 samples excluded, respectively), or showed any cryptic relations using KING cut-off of 0.177 (four and 51 samples excluded, respectively). To avoid potential confounding of results due to different genetic ancestries in the dataset, and to match with the genetic ancestries of the CKDGen dataset, non-European ancestry samples were excluded by using principal component analysis (PCA). This was computed using the The 1000 Genomes Project (1000GP), human genome build 38 (hg38) reference dataset and the plinkQC package in R, by adapting the published R script called “Processing 1000 Genomes reference data for ancestry estimation” on the plinkQC website (https://meyer-lab-cshl.github.io/plinkQC/articles/AncestryCheck.html) for hg38 use^61,62^. After combining the sample and reference datasets and running PCA, the plinkQC “evaluate_check_ancestry” function was used to select individuals of European descent^61^. This function uses principal components 1 and 2 to find the center of the known European ancestry reference samples. It then labels study samples as non-European if their Euclidean distance from the center falls outside the radius specified by the maximum Euclidean distance of the reference samples multiplied by a scaling factor. A scaling factor of 2 was chosen as it was the minimum value that produced a radius that included all the 1000GP European ancestry reference samples (Supplementary Figure 2). We identified a small number of 10 NURTuRE-CKD samples that self-declared as White British but were outside of the European ancestry reference radius using PCA (Supplementary Fig. 2). To avoid any potential confounding, these 10 were excluded as non-European based on the reference population. Overall, using this strategy, 350 NURTuRE-CKD, 19 NURTuRE-controls and 108 SKS non-European ancestry samples were excluded (Supplementary Fig. 3). Our adapted script is available here:

https://github.com/AmyJaneOsborne/CCA_scripts/PCA_ancestry_hg38_genotype_datasets.sh^61^. No batch effects were seen using PCA. Since CCA cannot handle any missing data, SNPs with any missing data were excluded. Remaining for analysis were 2505 of 2903 NURTuRE-CKD, 80 of 99 NURTuRE-controls and 2078 of 2409 SKS samples. For the NURTuRE-CKD (plus 80 NURTuRE-controls) dataset, 468,604 variants remained before SNP imputation. For the SKS dataset plus NURTuRE-controls dataset, 458,472 variants remained before SNP imputation. All calculations were performed in a 64-bit Linux conda environment.

### Kidney function data

For each participant, the first eGFR and serum urea measurements taken on the same date, either on exactly or on the closest after the cohort recruitment date were used. Of the 2505 European ancestry NURTuRE-CKD, 2078 SKS and 80 NURTURE-control samples, totals of 2475, 1898, and 38, respectively, had available eGFR and serum urea data on the same date. eGFR was calculated using the CKD-Epidemiology Collaboration (CKD-EPI) equation (without ethnicity adjustment). For NURTuRE-CKD, the ranges of the eGFR and serum urea values were 3.3–139 ml/min/1.73 m^2^ and 1.7–63.7 mmol/L, respectively. For NURTuRE-controls, the ranges of the eGFR and urea values were 53–95 ml/min/1.73 m^2^ and 3.5–8 mmol/L, respectively. For SKS, the ranges of the eGFR and urea values were 6–88 ml/min/1.73 m^2^ and 3–47.7 mmol/L, respectively. Urea was converted to BUN by dividing by a factor^63^. BUN measurements were standardized using the common log transformation and Z-score, and eGFR measurements were standardized using the rank-based inverse normal transformation, as described previously. In the CCA input data matrix, the CKD cases (*n* = 2475 and *n* = 1898, respectively) and controls (a different set of *n* = 19 for each dataset) were each encoded as binary indicator variables, 1 and 0, respectively. Kidney function variables were not adjusted for CKD status since they were correlated with CKD status and controlling for CKD status may have introduced collider bias.

### Single nucleotide polymorphism imputation

For the NURTuRE and SKS datasets, ungenotyped SNPs were imputed using Beagle version 5.4 with The 1000 Genomes Project hg38 genotype dataset as the reference dataset^64,65^. The 1000 Genomes Project hg38 Variant Call Format files were downloaded from the 1000 Genomes Project website (https://www.internationalgenome.org/data-portal/data-collection/grch38^64^. Imputation is based on the fact that physically close markers are likely inherited together in a cluster and therefore result in the non-random association of alleles (linkage disequilibrium). Imputation in Beagle is performed by using a Hidden Markov Model to identify the most likely path through the haplotype cluster based on the non-missing genotypes present^66^. The Beagle R^2^ accuracy score approximates the squared correlation between the best estimate genotype (i.e. the allele dosage with the highest posterior probability) and the true genotype^66^. This is estimated from the posterior genotype probabilities when the true genotypes are not observed^66^. Unix scripting was used to run Beagle and Plink commands. After imputation, there were 6,419,966 NURTuRE-CKD and 6,290,407 SKS variants for analysis (including the NURTuRE-controls in each dataset).

### Genome-wide association study summary statistics on kidney function

European ancestry CKDGen eGFR and BUN GWAS summary statistics were downloaded from the CKDGen Consortium website on 28/05/2020^17^. For eGFR and BUN, there were 567,460 samples including 41,395 CKD cases (7%), as described on their website (https://ckdgen.imbi.uni-freiburg.de/#Wuttke2019data)^17^.

Published BioBank Japan GWAS summary statistics (6,108,953 SNPs) for eGFR (bbj-a-60) and BUN (bbj-a-11) for 143,658 and 139,818 individuals, respectively, were downloaded from the Medical Research Council Integrative Epidemiology Unit OpenGWAS project website (https://gwas.mrcieu.ac.uk/datasets/bbj-a-60/) on 20/04/2021^16,67^. The East Asian ancestry dataset contained approximately 8586 CKD cases (5%). The eGFR range was 17.2–132.9 ml/min/1.73 m^2^^16,68^.

For each of the three GWAS summary statistics datasets, only SNPs in chromosomes 1–22 were selected since these were the only ones available in the CKDGen dataset. SNPs with both eGFR and BUN statistics available were selected based on matching chromosome, position, reference and effect alleles. For the CKDGen and BioBank Japan datasets, any SNPs with AF < 0.01 were excluded from the *metaCCA* analyses. For each dataset, 8,346,783 and 5,837,593 SNPs remained for analysis, respectively.

### Statistical analysis using canonical correlation analysis

CCA was used to compute canonical correlations between each SNP with both eGFR and BUN by using the “cancor” function in R v3.6.0, based on CCA scripts published by Seoane et al, Ferreira et al, and Tang et al.^23,25,26^. To analyze the NURTuRE and SKS datasets, we used our scripts available from Github (https://github.com/AmyJaneOsborne/CCA_scripts). In the CCA matrix, the same 26 controls were used for each of the NURTuRE and SKS dataset analyses. The controls were encoded as ‘0’ and the cases as ‘1’, as an additional binary indicator variable.

In CCA, linear combinations of two sets of variables, or views of the same object, X and Y, with the highest correlations are found. This corresponds to finding vectors ***a*** ∈ ℝ*G* and ***b*** ∈ ℝ*P* that maximize:

1 $${r}_{1}=\frac{{\left(X{{\boldsymbol{a}}}_{1}\right)}^{T}(Y{{\boldsymbol{b}}}_{1})}{{||X}{{\boldsymbol{a}}}_{1}{||\; ||Y}{{\boldsymbol{b}}}_{1}{||}}=\frac{{{\boldsymbol{a}}}_{1}^{T}{\sum }_{{XY}}{{\boldsymbol{b}}}_{1}}{\sqrt{{{\boldsymbol{a}}}_{1}^{T}{\sum }_{{XX}}{{\boldsymbol{a}}}_{1}}\sqrt{{{\boldsymbol{b}}}_{1}^{T}{\sum }_{{YY}}{{\boldsymbol{b}}}_{1}}}$$

This value ***r***_1_ is called (the first) *canonical correlation* between X and Y, and the corresponding vectors ***a***_1_ and ***b***_1_ are called (the first) canonical weights. To identify the variables that provide a large contribution to the observed canonical correlation, the magnitudes of the canonical weights are used. These are found by firstly computing the matrix *K* from the covariance matrices:

2 $$K={\sum }_{{XX}}^{-1/2}{\sum }_{{XY}}{\sum }_{{YY}}^{-1/2}.$$

Then, a Singular Value Decomposition (SVD) is applied, to decompose the matrix into eigenvectors and eigenvalues. The canonical correlation values can then be computed directly from the matrix *K*, and the canonical weights are determined based on the eigenvectors. To test the significance of each canonical correlation *r*, which equals the maximum correlation between the variant and the phenotypes, the test statistic was computed based on *Wilks’ Lambda*, as described previously^23^. For both CCA and *metaCCA*, for each test, the *N* parameter was set to the total number of samples analysed. Results were visualized using Manhattan plots. For *univariate*-SNP analyses, to account for multiple testing, the *P*-values were adjusted for multiple comparisons using a Bonferroni correction based on the number of SNPs analyzed. A cut-off of adjusted *P*-value < 0.05 was used to determine significance. For NURTuRE-CKD and SKS, our approximate power analysis based on univariate analysis suggested it was possible to identify a significant SNP with an effect size (canonical correlation *r*) of 0.12 or 0.14, respectively, with 80% power (Table 4). The sample size required to identify CCA correlation *r* of 0.1 with 90% power with two variables has been reported as approximately 1000 samples in Helmer et al.^69^

**Table 4:** Power analysis for two individual level genotype datasets

Dataset analysis	Participants (*n*)	Power	Significance level (Bonferroni-corrected)	r
NURTuRE-chronic kidney disease (CKD) and NURTuRE-controls, *univariate*-single nucleotide polymorphism	2494 including 19 controls	80%	5E−8	0.12
Salford Kidney Study (SKS) and NURTuRE-controls, *univariate*-single nucleotide polymorphism	1917 including 19 controls	80%	5E−8	0.14

### Statistical analysis using *metaCCA*

For each of the two GWAS summary statistics datasets, the *metaCcaGp* function provided by the *metaCCA* package in R v3.6.0 was used to compute the canonical correlation between each SNP and both eGFR and BUN^28,70^. Our scripts for analyzing the two GWAS summary statistics datasets using *metaCCA* are available from Github (https://github.com/AmyJaneOsborne/CCA_scripts)^28^. Before running *metaCCA*, SNPs were removed if they had a standard error of 0 for the eGFR or BUN beta coefficients, had reference and alternative alleles not containing ‘A’, ‘C’, ‘G’, or ‘T’, or if they were SNPs that were duplicates. A mathematical explanation of *metaCCA* is provided by Cichonska et al.^28^ After running *metaCCA*, any SNPs that showed a significant *metaCCA P*-value were assessed for kidney function relevance based on the published eGFR and BUN GWAS summary statistics. SNPs were excluded if the eGFR and BUN effect sizes (with respect to the same effect allele) were in the same direction, because they were unlikely to be relevant for kidney function as previously described^17^.

### Single nucleotide polymorphism annotation

SNPs were annotated and prioritized by using the Functional Mapping and Annotation of Genome-Wide Association Studies (FUMA) program^32^. FUMA “SNP2GENE” was used to find lead (most signficant, independent) SNPs for each locus genomic region, using default r^2^ thresholds of 0.6 to define independent significant SNPs and 0.1 to define lead SNPs^32^. The HUGO Gene Nomenclature Committee (HGNC) online multi-symbol checker was used to verify gene symbols^71^. g:Profiler g:GOSt and stringDB were used for functional enrichment analyses^72,73^. The effects of any missense SNPs were predicted by using four in silico variant prediction tools including FATHMM-XF, Combined Annotation Dependent Depletion (CADD), Sorting Intolerant From Tolerant (SIFT) and PolyPhen-2 (run by using the ensembl Variant Effect Predictor online tool)^74–77^. To test for significant overlap between dataset results, the hypergeometric test provided by the package “hypeR” (v1.5.4) via Bioconductor in R (v4.0.2) was used^78^.

### Colocalisation of CCA and eQTL signals

We applied Bayesian colocalisation analyses by using the R package ‘coloc’ (cran.r-project.org/web/packages/coloc)^33,34^. We applied the COLOC function, which uses Approximate Bayes Factor computations, to lead SNPs identified in CKDGen and BioBank Japan by *metaCCA*, and in NURTURE-CKD by CCA, and to overlapping lead SNPs identified across 2 or 3 datasets. We used default priors that a random variant in the region is associated with either kidney function *metaCCA*/CCA or eQTL individually (prior probabilities = 1 × 10^−4^), and set the prior probability that the random variant is causal to both kidney function *metaCCA*/CCA and eQTL (prior probability = 1 × 10^−6^). As recommended by the authors of the method, we defined the variants as colocalised when the posterior probability of a colocalised signal (PP4) was >0.8. We used all eQTL data available in the EBI eQTL Catalogue database which included Gtex v8 (https://www.ebi.ac.uk/eqtl/Data_access/). These were downloaded for analysis by adapting an R script available from the EBI public eQTL Catalogue resources (https://github.com/kauralasoo/eQTL-Catalogue-resources/blob/master/tutorials/tabix_use_case.html)^35^. We followed the coloc example in this script thus included the lead SNP plus surrounding SNPs within 200 kB as input. The single-SNP matrixEQTL results for NephQTL glomerular and tubular cells were downloaded from the NephQTL2 browser (https://hugeampkpn.org/research.html?pageid=nephqtl2_about_118)^79^.

### Allele frequency analyses

For any shortlisted SNPs that showed a difference of at least 10% in allele frequency between the gnomAD general population and each of the same CKD groups in NURTURE-CKD and SKS, a Chi-square test with Yates’ correction was used to test for significant association of the SNP with CKD cases^40^. This was analyzed using the online GraphPad QuickCalcs tool (https://www.graphpad.com/quickcalcs/contingency1/).

### Differential gene expression analyses

Using the Gene Expression Omnibus GEO2R application, the differential gene expression between CKD cases and healthy controls was computed for the “GSE66494: development of gene expression profiles in human chronic kidney disease” and “GSE37171: expression data from uremic patients and 20 healthy controls (normals)” datasets^37,38^. Uremia is the build-up of toxins in blood which occurs when the kidneys stop working and is a sign of CKD. The R scripts generated to reproduce the results are available from Github: https://github.com/AmyJaneOsborne/CCA_scripts. Genes were considered to show significant differential expression between the CKD cases and controls where the log2(fold change)≥1 or log2(fold change)≤−1 and adjusted *P*-value based on default Benjamini & Hochberg false discovery rate method < 0.05^39^.

### Reporting summary

Further information on research design is available in the Nature Research Reporting Summary linked to this article.

### Supplementary information

Supplementary Information

Reporting summary

Dataset 1

Dataset 2

Dataset 3

Dataset 4

Dataset 5

Dataset 6

Dataset 7

Dataset 8

Dataset 9

Dataset 10

Dataset 11

Dataset 12

Dataset 13

Dataset 14

Dataset 15

## Data Availability

The datasets analysed during the current study are available in the CKDGen Consortium repository (http://ckdgen.imbi.uni-freiburg.de/)^7^, The 1000 Genomes Project Phase 1 genotype repository (http://www.cog-genomics.org/plink/1.9/resources#1kg)^60^ and the EBI GWAS Catalog (https://www.ebi.ac.uk/gwas/studies/). Genotype-phenotype data access for NURTuRE-CKD is available by application to the Kidney Research UK NURTuRE Biobank resource (https://nurturebiobank.org/information-for-researchers/). Genotype-phenotype data access for the SKS is available by application to the Salford Kidney Study (https://www.hra.nhs.uk/planning-and-improving-research/application-summaries/research-summaries/salford-kidney-study/).

## Code availability

To analyze the NURTuRE-CKD dataset we used our scripts available from Github: https://github.com/AmyJaneOsborne/CCA_scripts, based on scripts used for our previous study^23^ using Canonical Correlation Analysis and also downloadable from Github: https://github.com/jseoane/gaCCA. Our scripts for analysing the three GWAS summary statistics datasets (see Methods) are available from Github: https://github.com/AmyJaneOsborne/CCA_scripts, and were based on *metaCCA*^28^.
