# Supplementary material for: Multivariate canonical correlation analysis identifies additional genetic variants for chronic kidney disease
Source: NPJ Syst Biol Appl. 2024 Mar 9;10:28. doi: 10.1038/s41540-024-00350-8 (PMC10924093; doi:10.1038/s41540-024-00350-8)
Supplement: Supplementary file 1 — Supplementary Information [file 41540_2024_350_MOESM1_ESM.pdf]

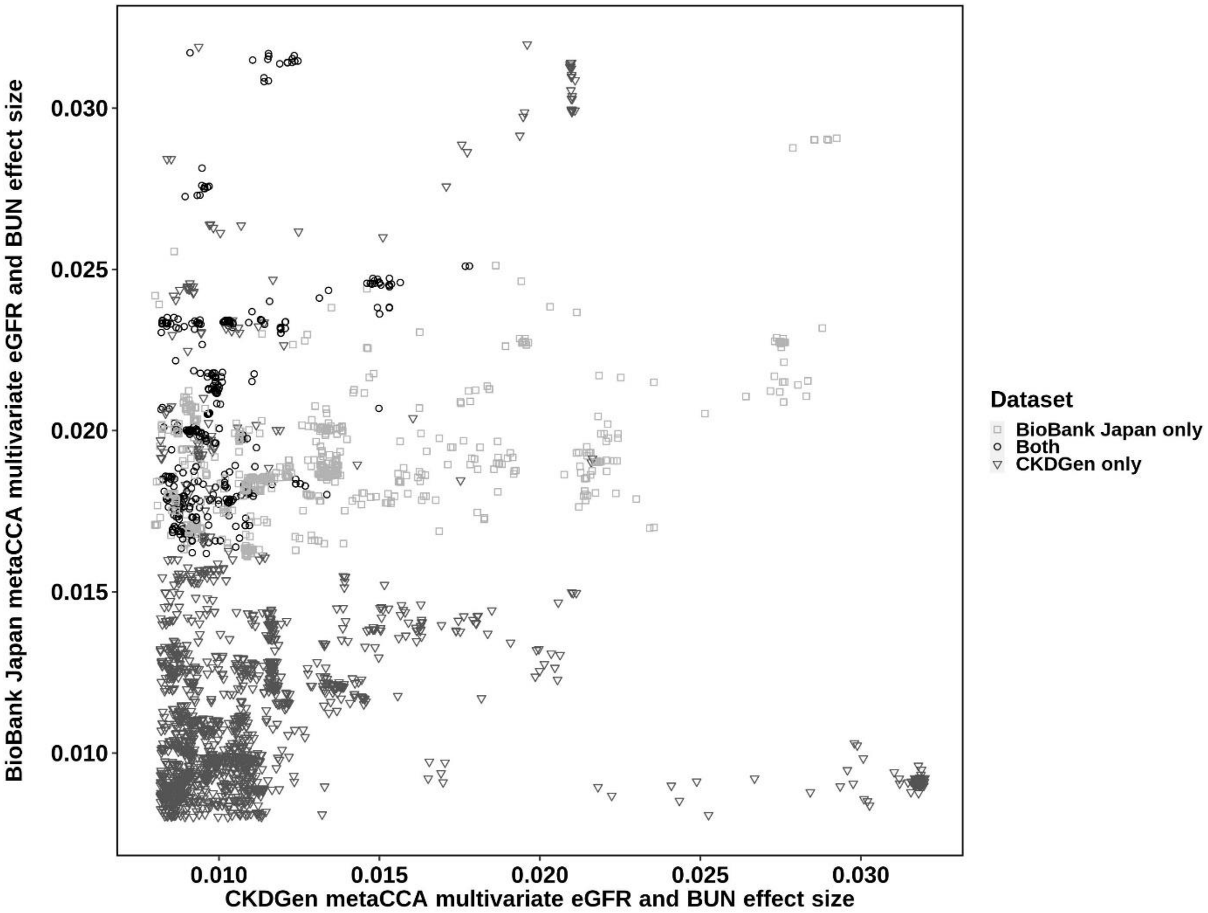

Supplementary Figure 1. *metaCCA* canonical correlation coefficients for previously unreported single nucleotide polymorphisms identified in both CKDGen and BioBank Japan. Shown are the multivariate canonical correlation coefficient effect sizes obtained for the previously unreported 5,840 CKDGen (dark grey triangular points) and 2,471 BioBank Japan (light grey square points) single nucleotide polymorphisms (SNPs), and their union of 394 SNPs in both datasets (black circle points), identified by *metaCCA* for both estimated glomerular filtration rate and blood urea nitrogen, jointly.

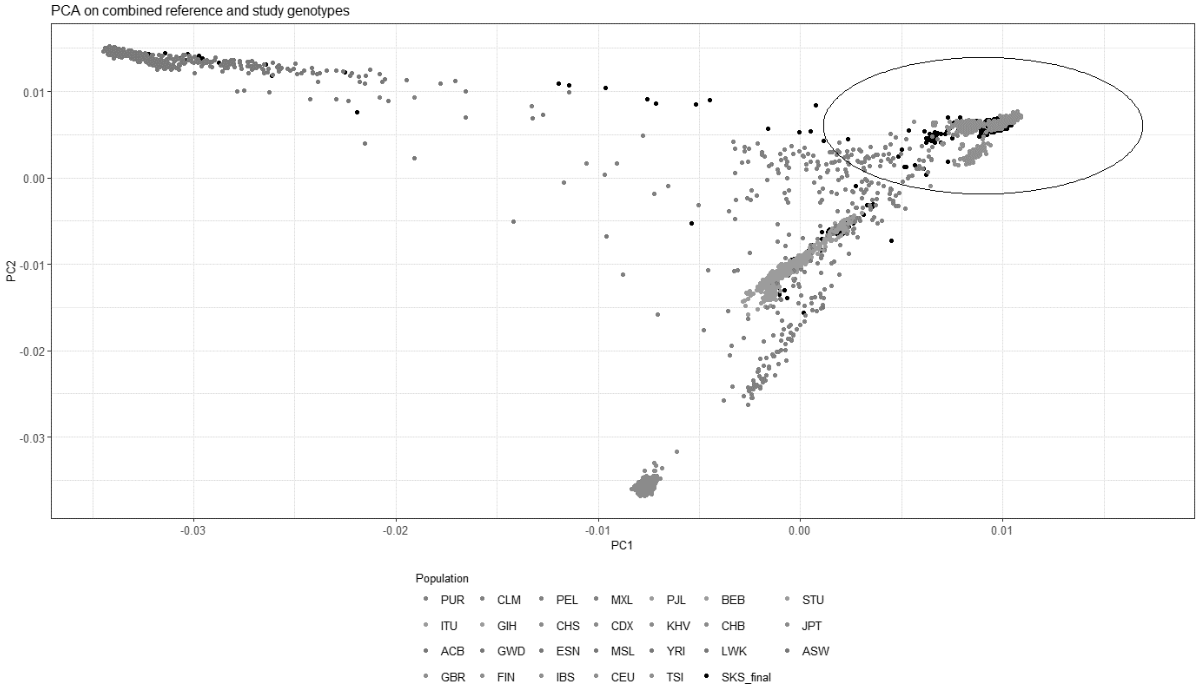

Supplementary Figure 2. Genetic ancestry analysis by principal components analysis for the NURTuRE-chronic kidney disease dataset. Principal components 1 and 2 of a principal components analysis for NURTuRE-CKD and The 1000 Genomes Project reference genotype datasets combined are shown. These components were used to find the centre of the known European ancestry reference samples. The NURTuRE-CKD samples (black points) within the circle, which marks the maximum Euclidean distance of the European ancestry reference samples from the centre and multiplied by a scaling factor of 2, were labelled as European ancestry. The remaining NURTuRE-CKD samples outside of the circle were labelled as non-European and excluded. African: African Caribbean (ACB), African Ancestry SW (ASW), Esan (ESN), Gambian Mandinka (GWD), Luhya (LWK), Mende (MSL), Yoruba (YRI). Central American: Colombian (CLM), Mexican Ancestry (MXL), Peruvian (PEL), Puerto Rican (PUR). East Asian: Dai Chinese (CDX), Han Chinese (CHB), Southern Han Chinese (CHS), Japanese (JPT), Kinh Vietnamese (KHV). European: Utah residents with Northern and Western European ancestry from the Center for the Study of Human Polymorphisms (CEU); Finnish (FIN), British (GBR), Iberian (IBS), Toscani (TSI). Blue points (South Asian): Bengali (BEB), Gujarati (GIH), Telugu (ITU), Punjabi (PJL), Tamil (STU).

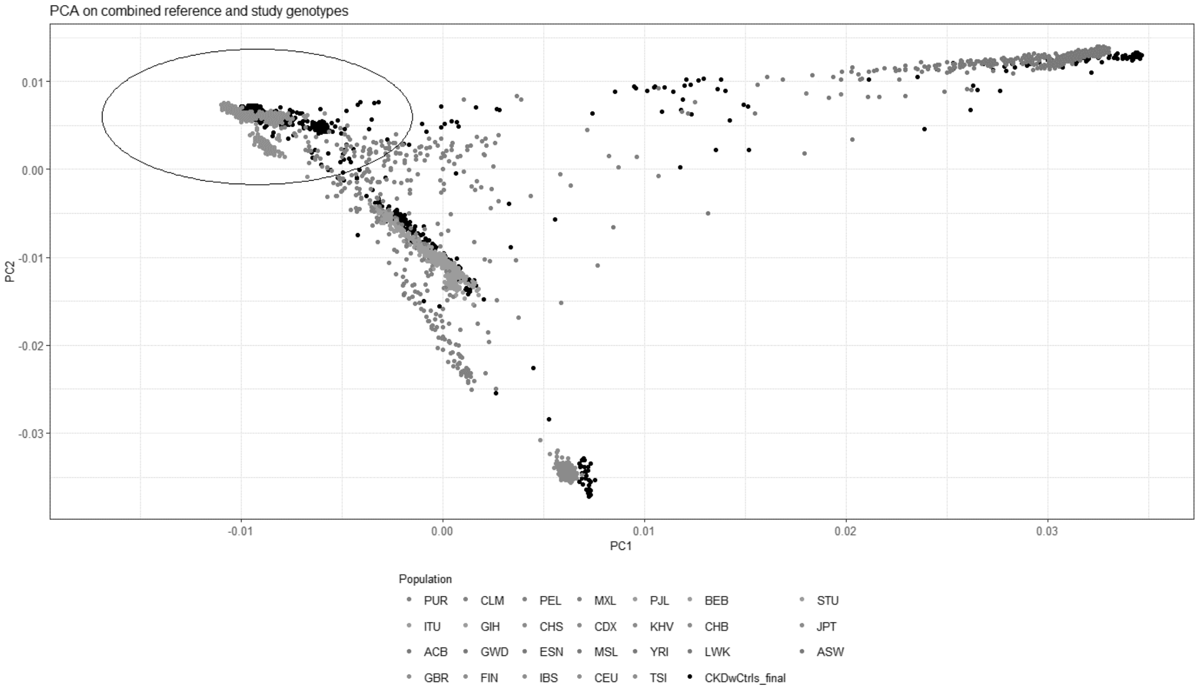

Supplementary Figure 3. Genetic ancestry analysis by principal components analysis for the Salford Kidney Study dataset. Principal components 1 and 2 of a principal components analysis for Salford Kidney Study (SKS) and The 1000 Genomes Project reference genotype datasets combined are shown. These components were used to find the centre of the known European ancestry reference samples (pink coloured points). The SKS samples (black points) within the black circle, which marks the maximum Euclidean distance of the European ancestry reference samples from the centre and multiplied by a scaling factor of 2, were labelled as European ancestry. The remaining SKS samples outside of the circle were labelled as non-European and excluded. Yellow points (African): African Caribbean (ACB), African Ancestry SW (ASW), Esan (ESN), Gambian Mandinka (GWD), Luhya (LWK), Mende (MSL), Yoruba (YRI). Green points (Central American): Colombian (CLM), Mexican Ancestry (MXL), Peruvian (PEL), Puerto Rican (PUR). Purple points (East Asian): Dai Chinese (CDX), Han Chinese (CHB), Southern Han Chinese (CHS), Japanese (JPT), Kinh Vietnamese (KHV). Pink points (European): Utah residents with Northern and Western European ancestry from the Center for the Study of Human Polymorphisms (CEU); Finnish (FIN), British (GBR), Iberian (IBS), Toscani (TSI). Blue points (South Asian): Bengali (BEB), Gujarati (GIH), Telugu (ITU), Punjabi (PJL), Tamil (STU).
