# Supplementary material for: Multivariate canonical correlation analysis identifies additional genetic variants for chronic kidney disease
Source: NPJ Syst Biol Appl. 2024 Mar 9;10:28. doi: 10.1038/s41540-024-00350-8 (PMC10924093; doi:10.1038/s41540-024-00350-8)
Supplement: Supplementary file 2 — Reporting summary [file 41540_2024_350_MOESM2_ESM.pdf]

nature portfolio

Corresponding author(s): Amy Osborne

Last updated by author(s): Dec 20, 2023

Reporting Summary

Nature Portfolio wishes to improve the reproducibility of the work that we publish. This form provides structure for consistency and transparency in reporting. For further information on Nature Portfolio policies, see our Editorial Policies and the Editorial Policy Checklist.

Statistics

For all statistical analyses, confirm that the following items are present in the figure legend, table legend, main text, or Methods section.

n/a	Confirmed	
☐	☒	The exact sample size (n) for each experimental group/condition, given as a discrete number and unit of measurement
☐	☒	A statement on whether measurements were taken from distinct samples or whether the same sample was measured repeatedly
☐	☒	The statistical test(s) used AND whether they are one- or two-sided<br>*Only common tests should be described solely by name; describe more complex techniques in the Methods section.*
☐	☒	A description of all covariates tested
☐	☒	A description of any assumptions or corrections, such as tests of normality and adjustment for multiple comparisons
☐	☒	A full description of the statistical parameters including central tendency (e.g. means) or other basic estimates (e.g. regression coefficient) AND variation (e.g. standard deviation) or associated estimates of uncertainty (e.g. confidence intervals)
☐	☒	For null hypothesis testing, the test statistic (e.g. F, t, r) with confidence intervals, effect sizes, degrees of freedom and P value noted<br>*Give P values as exact values whenever suitable.*
☐	☒	For Bayesian analysis, information on the choice of priors and Markov chain Monte Carlo settings
☒	☐	For hierarchical and complex designs, identification of the appropriate level for tests and full reporting of outcomes
☐	☒	Estimates of effect sizes (e.g. Cohen's d, Pearson's r), indicating how they were calculated

Our web collection on statistics for biologists contains articles on many of the points above.

Software and code

Policy information about availability of computer code

Data collection	n/a
Data analysis	- For genetic data processing: BCFTools version 1.9 (using htslib 1.9), Plink versions 1.9 and 2, the "plinkQC" package in R, and custom code at https://github.com/AmyJaneOsborne/CCA_scripts: PCA_ancestry_hg38_genotype_datasets.sh and gwas_qc_filters.sh.<br>- For single nucleotide polymorphism imputation: Beagle version 5.4.<br>- For canonical correlation analysis and metaCCA: "cancor" function and metaCCA package in R v3.6.0 and custom code at https://github.com/AmyJaneOsborne/CCA_scripts.<br>- For lead single nucleotide polymorphism analysis: Functional Mapping and Annotation of Genome-Wide Association Studies (FUMA) "SNP2GENE".<br>- HUGO Gene Nomenclature Committee (HGNC) online multi-symbol checker was used to verify gene symbols.<br>- g:Profiler g:GOSt (https://biit.cs.ut.ee/gprofiler/) was used for functional enrichment analyses.<br>- Missense SNP effect predictions by four in silico variant prediction tools: FATHMM-XF, Combined Annotation Dependent Depletion (CADD), Sorting Intolerant From Tolerant (SIFT) and PolyPhen-2 (run by using the ensembl Variant Effect Predictor online tool).<br>- To test for significant overlap between dataset results, the hypergeometric test provided by the package "hypeR" (v1.5.4) via Bioconductor in R (v4.0.2) was used.<br>- We applied Bayesian colocalisation analyses by using the R package 'coloc' . These were downloaded for analysis by adapting an R script available from the EBI public eQTL Catalogue resources (https://github.com/kauralasoo/eQTL-Catalogue-resources/blob/master/tutorials/tabix_use_case.html).<br>- Statistical allele frequency analyses were carried out using the online GraphPad QuickCalcs tool (https://www.graphpad.com/quickcalcs/contingency1/).

April 2023

- Gene Expression Omnibus GEO2R application was used for differential gene expression analysis.
- Data visualisation using various packages in R (ggplot2, grid, sqldf, dplyr, scales, stringr, tidyr, ggbreak)

For manuscripts utilizing custom algorithms or software that are central to the research but not yet described in published literature, software must be made available to editors and reviewers. We strongly encourage code deposition in a community repository (e.g. GitHub). See the Nature Portfolio guidelines for submitting code & software for further information.

Data

Policy information about availability of data

All manuscripts must include a data availability statement. This statement should provide the following information, where applicable:

- Accession codes, unique identifiers, or web links for publicly available datasets
- A description of any restrictions on data availability
- For clinical datasets or third party data, please ensure that the statement adheres to our policy

The datasets analysed during the current study are available in the CKDGen Consortium repository (http://ckdgen.imbi.uni-freiburg.de/),8 The 1000 Genomes Project Phase 1 genotype repository (http://www.cog-genomics.org/plink/1.9/resources#1kg)78 and the EBI GWAS Catalog (https://www.ebi.ac.uk/gwas/studies/). Genotype-phenotype data access for NURTuRE-CKD is available by application to the NURTuRE biobank (https://www.nurturebiobank.org/about/). Genotype-phenotype data access for the SKS is available by application to the Salford Kidney Study (https://www.hra.nhs.uk/planning-and-improving-research/application-summaries/research-summaries/salford-kidney-study/).

Research involving human participants, their data, or biological material

Policy information about studies with human participants or human data. See also policy information about sex, gender (identity/presentation), and sexual orientation and race, ethnicity and racism.

Reporting on sex and gender	n/a
Reporting on race, ethnicity, or other socially relevant groupings	For the NURTURE-CKD and SKS genetic variant datasets, to avoid potential confounding of results due to different genetic ancestries in the dataset, and to match with the genetic ancestries of the CKDGen dataset, non-European ancestry samples were excluded by using principal component analysis (PCA). This was computed using the The 1000 Genomes Project (1000GP), human genome build 38 (hg38) reference dataset and the plinkQC package in R, by adapting the published R script called “Processing 1000 Genomes reference data for ancestry estimation” on the plinkQC website (https://meyer-lab-cshl.github.io/plinkQC/articles/AncestryCheck.html) for hg38 use.
Population characteristics	n/a
Recruitment	Described in the original studies for the NURTURE-CKD and SKS datasets:<br>- Taal, M. W. et al. Associations with age and glomerular filtration rate in a referred population with chronic kidney disease: Methods and baseline data from a UK multicentre cohort study (NURTuRE-CKD). Nephrol Dial Transplant, doi:10.1093/ndt/gfad110 (2023).<br>- Ali, I., Donne, R. L. & Kalra, P. A. A validation study of the kidney failure risk equation in advanced chronic kidney disease according to disease aetiology with evaluation of discrimination, calibration and clinical utility. BMC Nephrol 22, 194, doi:10.1186/s12882-021-02402-1 (2021).
Ethics oversight	The NURTuRE-CKD cohort study was approved by the South Central—Berkshire Research Ethics Committee.<br>The Salford Kidney Study (SKS) dataset received ethical approval from the North West Greater Manchester South Research Ethics Committee.

Note that full information on the approval of the study protocol must also be provided in the manuscript.

Field-specific reporting

Please select the one below that is the best fit for your research. If you are not sure, read the appropriate sections before making your selection.

☒ Life sciences ☐ Behavioural & social sciences ☐ Ecological, evolutionary & environmental sciences

For a reference copy of the document with all sections, see nature.com/documents/nr-reporting-summary-flat.pdf

Life sciences study design

All studies must disclose on these points even when the disclosure is negative.

Sample size	For NURTuRE-CKD and SKS, our power analysis based on the numbers of participants in each study and for univariate analysis suggested it was possible to identify a significant SNP with an effect size (canonical correlation r) of 0.12 or 0.14, respectively, with 80% power (Table 4). The sample size required to identify CCA correlation r of 0.1 with 90% power with two variables has been reported as approximately 1000 samples in Supplementary Figure 15A of Helmer, M. et al. On stability of Canonical Correlation Analysis and Partial Least Squares with application to brain-behavior associations. bioRxiv, 2020.2008.2025.265546, doi:10.1101/2020.08.25.265546 (2023).
Data exclusions	Standard genome-wide SNP array exclusions were applied as follows. For each of the NURTURE-CKD and SKS datasets, variants were excluded

Data exclusions	if the minor allele frequency (MAF) < 0.01 (162,483 and 170,554 variants excluded, respectively), missing SNP genotype call rate >= 1.5% (19,149 and 23,889 SNPs excluded, respectively), Hardy-Weinberg assumptions violated with P-value < 0.001% (116 and 71 SNPs excluded, respectively) and if they were located on mitochondrial or sex chromosomes (21,133 and 19,426 SNPs excluded, respectively). From the SKS dataset only, a total of 135 overlapping samples with NURTURE-CKD were excluded. Further samples were excluded if they were known first or second degree relatives of another participant (13 and 0 excluded), showed gender mismatches (11 and 33 excluded), showed >10% low call rate SNPs (12 and 32 samples excluded, respectively), or showed any cryptic relations using KING cut-off of 0.177 (four and 51 samples excluded, respectively). To avoid potential confounding of results due to different genetic ancestries in the dataset, and to match with the genetic ancestries of the CKDGen dataset, non-European ancestry samples were excluded by using principal component analysis (PCA). Since canonical correlation analysis cannot handle any missing data, SNPs with any missing data were excluded.
Replication	1. Of the potentially novel 5,840 CKDGen and 2,471 BioBank Japan SNPs for kidney function we identified by metaCCA, 4,855 and 2,091 SNPs, respectively, were available in both datasets, and of these, there was an overlap (replication) of 394 (8% and 19%, respectively) SNPs. This overlap of 394 replicated SNPs was significant compared to that expected for the number of SNPs analysed using the hypergeometric distribution (P-value < 0.05; Table 2).<br><br>2. We investigated whether any of the CKDGen metaCCA-identified SNPs that showed eGFR and BUN effect sizes in opposite directions (14,045 of 26,562 SNPs) showed replicated kidney function associations in each of the NURTuRE-CKD (n = 2,494 including 19 healthy participants) and SKS (n = 1,917 including a different set of 19 healthy participants) individual-level SNP genotype datasets by using CCA (Figure 1). Of these 14,045 metaCCA-identified SNPs, 12,711 SNPs were available for analysis in both the NURTURE-CKD and SKS datasets (Table 2). Of these 12,711 SNPs, 62 (0.5%) SNPs showed nominally significant CCA correlation with both eGFR and BUN in both the NURTuRE-CKD and SKS datasets (P-value < 0.05; Table 2, Figure 5B). This overlap of 62 metaCCA or CCA-identified SNPs replicated between the CKDGen (12,711 SNPs), NURTuRE-CKD (272,655) and SKS (268,630) datasets was statistically significant compared to that expected by chance for the number of SNPs analysed using the hypergeometric distribution (P-value < 0.05; Table 2).
Randomization	No experimental groups allocated.
Blinding	No group allocations needed.

Reporting for specific materials, systems and methods

We require information from authors about some types of materials, experimental systems and methods used in many studies. Here, indicate whether each material, system or method listed is relevant to your study. If you are not sure if a list item applies to your research, read the appropriate section before selecting a response.

Materials & experimental systems

n/a	Involved in the study
☒	☐ Antibodies
☒	☐ Eukaryotic cell lines
☒	☐ Palaeontology and archaeology
☒	☐ Animals and other organisms
☐	☒ Clinical data
☒	☐ Dual use research of concern
☒	☐ Plants

Methods

n/a	Involved in the study
☒	☐ ChIP-seq
☒	☐ Flow cytometry
☒	☐ MRI-based neuroimaging

Clinical data

Policy information about clinical studies

All manuscripts should comply with the ICMJE guidelines for publication of clinical research and a completed CONSORT checklist must be included with all submissions.

Clinical trial registration	For NURTURE-CKD (NCT04084145), see: Taal, M. W. et al. Associations with age and glomerular filtration rate in a referred population with chronic kidney disease: Methods and baseline data from a UK multicentre cohort study (NURTuRE-CKD). Nephrol Dial Transplant, doi:10.1093/ndt/gfad110 (2023).<br>For SKS, see: Ali, I., Donne, R. L. & Kalra, P. A. A validation study of the kidney failure risk equation in advanced chronic kidney disease according to disease aetiology with evaluation of discrimination, calibration and clinical utility. BMC Nephrol 22, 194, doi:10.1186/s12882-021-02402-1 (2021).
Study protocol	For NURTURE-CKD, see: Taal, M. W. et al. Associations with age and glomerular filtration rate in a referred population with chronic kidney disease: Methods and baseline data from a UK multicentre cohort study (NURTuRE-CKD). Nephrol Dial Transplant, doi:10.1093/ndt/gfad110 (2023).<br>For SKS, see: Ali, I., Donne, R. L. & Kalra, P. A. A validation study of the kidney failure risk equation in advanced chronic kidney disease according to disease aetiology with evaluation of discrimination, calibration and clinical utility. BMC Nephrol 22, 194, doi:10.1186/s12882-021-02402-1 (2021).
Data collection	For NURTURE-CKD, see: Taal, M. W. et al. Associations with age and glomerular filtration rate in a referred population with chronic kidney disease: Methods and baseline data from a UK multicentre cohort study (NURTuRE-CKD). Nephrol Dial Transplant, doi:10.1093/ndt/gfad110 (2023).<br>For SKS, see: Ali, I., Donne, R. L. & Kalra, P. A. A validation study of the kidney failure risk equation in advanced chronic kidney disease

	according to disease aetiology with evaluation of discrimination, calibration and clinical utility. BMC Nephrol 22, 194, doi:10.1186/s12882-021-02402-1 (2021).
Outcomes	n/a

Plants

Seed stocks	n/a
Novel plant genotypes	n/a
Authentication	n/a
